# Novel *O*-alkyl Derivatives of Naringenin and Their Oximes with Antimicrobial and Anticancer Activity

**DOI:** 10.3390/molecules24040679

**Published:** 2019-02-14

**Authors:** Joanna Kozłowska, Ewa Grela, Dagmara Baczyńska, Agnieszka Grabowiecka, Mirosław Anioł

**Affiliations:** 1 Department of Chemistry, Faculty of Biotechnology and Food Science, Wrocław University of Environmental and Life Sciences, Norwida 25, 50-375 Wrocław, Poland; miroslaw.aniol@upwr.edu.pl; 2 Department of Bioorganic Chemistry, Faculty of Chemistry, Wrocław University of Technology, Wybrzeże Wyspiańskiego 27, 50-370 Wrocław, Poland; ewa.grela@pwr.edu.pl (E.G.); agnieszka.grabowiecka@pwr.edu.pl (A.G.); 3 Department of Molecular and Cellular Biology, Faculty of Pharmacy with Division of Laboratory Diagnostics, Wroclaw Medical University, Borowska 211A, 50-556 Wrocław, Poland; dagmara.baczynska@umed.wroc.pl

**Keywords:** naringenin, *O*-alkyl derivatives, oximes, antibacterial activity, anticancer activity

## Abstract

In our investigation, we concentrated on naringenin (**NG**)—a widely studied flavanone that occurs in citrus fruits. As a result of a reaction with a range of alkyl iodides, 7 novel *O*-alkyl derivatives of naringenin (**7a**–**11a**, **13a**, **17a**) were obtained. Another chemical modification led to 9 oximes of *O*-alkyl naringenin derivatives (**7b**–**13b**, **16b**–**17b**) that were never described before. The obtained compounds were evaluated for their potential antibacterial activity against *Escherichia coli*, *Staphylococcus aureus*, and *Bacillus subtilis*. The results were reported as the standard minimal inhibitory concentration (MIC) values and compared with naringenin and its known *O*-alkyl derivatives. Compounds **4a**, **10a**, **12a**, **14a**, **4b**, **10b**, **11b**, and **14b** were described with MIC of 25 µg/mL or lower. The strongest bacteriostatic activity was observed for 7-*O*-butylnaringenin (**12a**) against *S. aureus* (MIC = 6.25 µg/mL). Moreover, the antitumor effect of flavonoids was examined on human colon cancer cell line HT-29. Twenty-six compounds were characterized as possessing an antiproliferative activity stronger than that of naringenin. The replacement of the carbonyl group with an oxime moiety significantly increased the anticancer properties. The IC_50_ values below 5 µg/mL were demonstrated for four oxime derivatives (**8b**, **11b**, **13b** and **16b**).

## 1. Introduction

About half of pharmacologically active compounds introduced to the market originate from natural biogenic molecules [1]. The search for novel drug candidates, especially of natural origin, maintains an unflagging interest in the scientific community. Among them, flavonoids have gained substantial popularity. They belong to polyphenolic compounds ubiquitous in photosynthesizing cells. Despite the structural homogeneity of the group, flavonoids play a wide variety of functions in plants, where they are responsible for bright coloring, cell protection, photosensitization, growth regulation, etc. Flavonoids also interact with mammalian and microbial cells. This versatility of biological function can be explained as the result of the interaction of cell enzymes with different structural parts of the flavonoid molecule, e.g., carbohydrate or phenyl ring. The wide occurrence of flavonoids in plants implies their importance in the human diet, as they provide important pro-health properties, usually coupled with minimal toxicity [2]. Currently, flavonoids are present on the consumer market as dietary supplements or their constituents.

Naringenin (4′,5,7-trihydroxyflavanone) is a well characterized flavonoid, with biological properties representative of this group of compounds. Its antimicrobial [2,3], anti-inflammatory, antioxidant [4], and anticancer [5] activity is well-described in the scientific literature. These desirable qualities of naringenin are coupled with a low cytotoxic effect on normal cell lines [6]. In nature, naringenin occurs mostly in its glycoside form—naringin, found primarily in citrus fruits such as grapefruits and sour oranges. The bitter taste of citrus fruits is related to the presence of naringin in peel, seeds, and pulp [7,8]. In the human digestive system naringin is easily hydrolyzed by bacterial microflora to its active aglycone—naringenin [9,10]. In addition, the base source of naringin defines the ease of its extraction from food industry waste products.

The valuable biological properties of naringenin can be further enhanced by its modification with a variety of active groups, attached mainly in the C-7 and C-4′ positions of the molecule. So far, a variety of *O*-alkyl naringenin derivatives have been described, both synthetic and of natural origin. Sakuranetin (7-*O*-methylnaringenin), a natural phytoalexin in rice [11], occurring also in kino extracts from *Corymbia torelliana* (Myrtaceae), was characterized as exhibiting antimicrobial activity against Gram-negative and Gram-positive bacteria in vitro [12]. Brazilian scientists isolated sakuranetin from twigs of *Baccharis retusa* (Asteraceae) and investigated its effectiveness in a murine model of experimental asthma. Sakuranetin was described as possessing antioxidant and anti-inflammatory properties and prevented vascular and distal parenchyma changes, which often contribute to pulmonary alterations observed in asthmatic patients [13]. Another ether derivative of naringenin that occurs in plant extracts of the Boraginaceae family, is 7,4′-di-*O*-methylnaringenin [14]. Recent reports showed that derivatives of naringenin with methyl groups attached to the C-7 and C-4′ positions exhibited a strong anti-seizures effect in a larval zebrafish model. Thus, sakuranetin and 7,4′-di-*O*-methylnaringenin were proposed as agents capable of attenuating epileptic attacks [15]. Another derivative, 7-*O*-butylnaringenin was described as a potent antibacterial agent in the treatment of infections caused by various common pathogens, such as methicillin-resistant *Staphylococcus aureus* (MRSA) [16] and *Helicobacter pylori* [17]. 7-*O*-Butylnaringenin also demonstrated a high cytotoxic effect on human breast cancer cell line MCF-7, supreme to that of naringenin [18]. In our previous work we described the synthesis and antimicrobial activity of *O*-methyl, *O*-ethyl, *O*-pentyl and *O*-dodecyl naringenin derivatives (**1a**–**6a**, **14a**–**15a**, **18a**–**19a**, **1b**–**6b**, **14b**–**15b**, **18b**–**19b**) [19]. 7,4′-Di-*O*-pentylnaringenin (**15a**) and 7-*O*-dodecylnaringenin (**18a**) were characterized as exhibiting the strongest antibacterial activity, which completely restricted the growth of *E. coli* strain at a concentration of 0.1% (*w*/*v*).

Alongside the modification of naringenin with *O*-alkyl chains, the incorporation of an oxime group was observed to significantly increase its biological activity. In many cases, the replacement of the carbonyl group with an oxime at the C-4 position resulted in higher antibacterial [20] and cytotoxic activities [21]. The modification of naringenin to its oxime enhanced its antitumor activity against rat pheochromocytoma (PC-12), human colon (HT-29) and breast (MCF-7) cancer cell lines [6]. The incorporation of an oxime group in *Kaempferia parviflora* flavonoids was coupled with a ‘dramatic improvement’ in their cytotoxicity against human epidermoid carcinoma of oral cavity (KB) and human small cell lung (NCI-H187) cancer cell lines [21]. Another flavonoid with similar structure to naringenin–chrysin (5,7-dihydroxyflavone) occurs naturally in honey, propolis, and a variety of plants e.g. passion fruits. Current literature reports, that the incorporation of methyl substituents at the C-5 and the C-7 positions of chrysin enhanced its anticancer activity against human gastric (SGC-7901) and human colon (HT-29) cell lines [22]. Furthermore, selenium and tellurium-derivatives of chrysin exhibited high antioxidant [23] and anticancer activity against lung adenocarcinoma (A-549) cells [24], comparing to inactive, unmodified 5,7-dihydroxyflavone.

In this paper, we present the efficient synthesis of 18 compounds, including 7 novel *O*-alkyl naringenin derivatives (**7a**–**11a**, **13a**, **17a**) and 9 new oximes (**7b**–**13b**, **16b**–**17b**), that have never been described in the scientific literature. All newly synthetized and previously described [19] naringenin derivatives (**1a**–**19a**, **1b**–**19b**) were evaluated for their bacteriostatic activity—the minimal inhibitory concentration (MIC) values were calculated for three bacterial strains: *Escherichia coli*, *Bacillus subtilis*, and *Staphylococcus aureus*. Moreover, the cytotoxic effect of naringenin derivatives was assayed using human colon cancer cell line HT-29.

## 2. Results and Discussion

### 2.1. Chemistry

**Figure 1:**
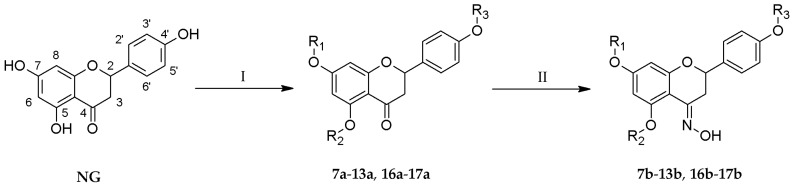
Synthesis of novel *O*-alkyl derivatives **7a**–**13a** and **16a**–**17a** and their oximes **7b**–**13b** and **16b**–**17b**; Reaction conditions: (I) alkyl iodide, (CH_3_)_2_CO or DMF, K_2_CO_3_, 40–45 °C, 40–96 h; (II) NH_2_OH·HCl, CH_3_COONa, EtOH, 40–50 °C, 24–96 h.

One-step reactions of naringenin with a variety of alkyl iodides allowed us to obtain novel *O*-alkyl derivatives (**7a**–**11a**, **13a**, **17a**) (Table 1). Reactions performed in anhydrous acetone yielded 7-*O*-alkyl- (**7a**, **10a**, **12a**, **16a**) and 7,4′-di-*O*-alkyl derivatives of naringenin (**8a**, **11a**, **13a**, **17a**) (Figure 1). The change to another organic solvent—*N*,*N*-dimethylformamide—enabled the formation of 5,7,4′-tri-*O*-propylnaringenin (**9a**) in a short period of time. Subsequently, all naringenin analogues were used as substrates in the reaction with hydroxylamine hydrochloride and anhydrous sodium acetate, dissolved in ethanol, which afforded novel oximes of *O*-alkyl derivatives (**7b**–**13b**, **16b**–**17b**). The crude products were purified by column chromatography.

The structures of the compounds were characterized by nuclear magnetic resonance (NMR) and high-resolution mass spectrometry (HRMS). The attachment of propyl, isopropyl, butyl, and decyl groups to hydroxyl moieties at the C-4′, C-7, and C-5 positions was confirmed by the analysis of the signals in ^1^H-NMR spectra (**7a**–**13a**, **16a**–**17a**).

In the case of 7-*O*-alkyl derivatives, singlets at 12.02–12.00 ppm and at 5.68–5.20 ppm described the presence of hydroxyl groups at the C-5 and C-4′ positions, respectively. Furthermore, the substitution of *O*-alkyl chains at the C-7 and C-4′ positions of the naringenin molecule was confirmed by the presence of only one singlet at 12.02–12.01 ppm, originating from the hydroxyl group at the C-5 position. The regioselectivity of the above alkylation reaction of naringenin was generally described in scientific reports [25,26]. In our investigation, one novel tri-*O*-alkyl derivative was obtained, namely 5,7,4′-tri-*O*-propylnaringenin (**9a**). The low reactivity of the hydroxyl moiety at the C-5 position is connected with the formation of an intramolecular hydrogen bond with the carbonyl group [27]. It was also visible in the ^1^H-NMR spectrum as a shift of the proton signal of the hydroxyl group at 12.02–12.01 ppm. Moreover, characteristic doublets of doublets from H-2, H-3_a_, and H-3_b_ provided the information about the flavanone skeleton of each derivative. The analysis of the signals in the ^13^C-NMR spectra at 196.24–189.34 ppm proved the presence of a carbonyl group.

In the case of oxime derivatives (**7b**–**13b**, **16b**–**17b**), the downshift to 154.86–147.77 ppm in the ^13^C-NMR indicated the replacement of the carbonyl group with the oxime moiety. In addition, a peak at 11.02–10.81 ppm in the ^1^H-NMR spectra proved the presence of the =NOH group in each product.

### 2.2. Antibacterial Activity

The growing resistance of pathogenic strains to popular antibiotics encourages the search for novel antimicrobials [28]. Compounds of natural origin represent a promising group with antibacterial, antifungal, and antiviral activities [2,29]. Flavonoids, often described as a protective barrier of plants [30], have been thoroughly examined for their advantageous properties. In our recent paper, a group of active naringenin derivatives was described [19]. Although naringenin itself did not affect bacterial growth, the incorporation of an aliphatic chain into the molecule positively altered its antibacterial characteristics [3,19]. In the presented research, we pursued evaluation of the antibacterial characteristics of 16 novel naringenin derivatives and compared them with the activity of other compounds possessing a similar structure, mostly synthesized and described for the first time by our research group [19]. The antibacterial activity was tested against three bacterial strains, Gram-negative *E. coli* and Gram-positive *B. subtilis* and *S. aureus*. The starting compound—naringenin (**NG**)—was characterized as exhibiting moderate antibacterial activity against both tested Gram-positive strains with MIC of 200 µg/mL each but was ineffective against *E. coli* up to a concentration of 400 µg/mL (Table 2). The lower susceptibility of Gram-negative strains to flavonoids has been described before and explained as the effect of the presence of a bacterial outer-membrane, especially the lipopolysaccharides barrier function [2]. This trend was further confirmed in the presented research, as most of the examined compounds were inactive or of low activity against *E. coli*. Among 39 tested naringenin derivatives, six inhibited the growth of *E. coli* with MIC of 200 µg/mL, namely, **2a**, **14a**, **12b**–**15b**. In contrast to the susceptibility of Gram-positive strains, presented in a later section of this paper, the growth of Gram-negative bacteria was limited by both mono- and di-*O*-alkyl derivatives.

A group of obtained naringenin derivatives was characterized as having a high bacteriostatic activity against both tested Gram-positive strains. Eight compounds were described with MIC of 25 µg/mL or lower (Table 2). The strongest activity was observed for 7-*O*-butylnaringenin (**12a**, MIC = 6.25 µg/mL against *B. subtilis* and *S. aureus*) and *O*-isopropyl derivatives (**10a**, **10b** and **11b**, MIC = 12.5 µg/mL against *B. subtilis* and *S. aureus*). In comparison to the starting compound, naringenin (**NG**, MIC = 200 μg/mL against *B. subtilis* and *S. aureus*), a significant improvement in the bacteriostatic activity was achieved. Although MIC values of the most potent derivatives (**10a**, **10b**, **11b**, **12a**) assayed against Gram-positive strains were an order of magnitude weaker than that of the antibiotics, gentamicin and novobiocin, their activity was comparable to the other positive control, nalidixic acid, against *E. coli* (MIC = 6.25 µg/mL).

In contrast to other reports [3,32], further elongation of aliphatic chain attached to the hydroxyl group at the C-7 position did not enhance the antibacterial properties. Generally, derivatives with C_10_ and C_12_ substituents were characterized with a low activity in comparison to that of compounds with shorter aliphatic chains. Additionally, the incorporation of more than one *O*-alkyl chain drastically decreased the antibacterial activity of the examined flavonoids against both Gram-positive strains used in the study. Surprisingly, the additional incorporation of the oxime group did not always result in a stronger antibacterial activity. The majority of active compounds shared the same values of MIC between *O*-alkyl derivatives and their corresponding oximes (e.g. **4a** and **4b** with MIC = 25 µg/mL against Gram-positive bacteria). This observation remains in contrast to other known reports [21,33], which indicated a higher antiproliferative activity of oxime derivatives.

According to the literature, the antibacterial mechanism of flavonoids is not uniform. Usually, more than a single site of action is suggested, as these structures may inhibit the synthesis of nucleic acids, impair cytoplasmic membrane function, or even affect energy metabolism [32]. The antibacterial activity of naringenin is most often coupled with its ability to interfere with bacterial cell membranes. Depending on the concentration and conditions of incubation a variety of effects may be observed. The reduction of membrane fluidity, bacteria permeabilization or even cell fusion are possible [2]. This mechanism is supported by the observation that the antibacterial activity may be further enhanced with hydrophobic substituents attached to flavonoid rings [32]. Naringenin (as opposed to naringin) was also proved to interfere with bacterial quorum-sensing mechanisms [34]. While it is a desirable factor, especially for compounds of medical use, it does not affect bacterial growth under standard in vitro conditions. Therefore, it can be assumed that bacteriostatic activity observed for the examined naringenin derivatives is caused by their interaction with bacterial cell membranes.

### 2.3. Anticancer Activity

The antitumor potential of flavonoids coupled with their low toxicity on normal cell lines has been studied for a long period of time. The possibility of their versatile modifications enables the synthesis of potential anticancer agents with enhanced bioavailability. It was already shown that *O*-alkyl derivatives may be supreme to compounds with unprotected hydroxyl groups [27,32]. Natural flavonoids from *Kaempferia parviflora* that include a variety of *O*-methyl derivatives of flavone may serve as an example [21]. Namely, 5,7-dimethoxyflavone exhibited a stronger chemopreventive activity than that of 5,7-dihydroxyflavone–chrysin. In addition, flavonoid modifications with oxime groups create a possibility of strengthening their cytotoxic properties. Naringenin itself expresses an anticancer activity that can be further enhanced with the incorporation of the oxime group at the C-4 position of the molecule [6]. There are also other possible substitutions, e.g., with aliphatic chains, that increase the anticancer properties of the molecule [35]. In our study numerous potential anticancer agents were found among the tested *O*-alkyl naringenin derivatives. Cytotoxicity evaluated on human colon adenocarcinoma HT-29 cell line pointed out the supremacy of the obtained oxime derivatives compared with the other examined compounds (Table 3).

Among the tested flavonoids, 14 compounds were described with IC_50_ values below 10 μg/mL: three of them being *O*-alkyl derivatives and the remaining 11 being oximes (Table 3). The lowest IC_50_ values were found among the investigated oximes. Compounds with double *O*-propyl, *O*-isopropyl, and *O*-butyl substituents at the C-7 and C-4′ positions (**8b**, **11b**, **13b**) and with *O*-decyl group at the C-7 position (**16b**) yielded an IC_50_ value 5 μg/mL or less. All these compounds were superior to their corresponding *O*-alkyl derivatives. Contrarily, the presence of =N-OH group instead of C=O in 5,7,4′-tri-*O*-alkylnaringenin derivatives containing *O*-methyl, *O*-ethyl, and *O*-propyl substituents resulted in decrease of their anticancer activity. Similar effect was observed for the derivatives containing *O*-decyl (**17a**, **17b**) and *O*-dodecyl (**19a**, **19b**) groups at the C-7 and C-4′ positions. The incorporation of the oxime group into the inactive 7,4′-di-*O*-propylnaringenin (**8a**, IC_50_ > 100 µg/mL) allowed us to obtain a highly potent antiproliferative agent—7,4′-di-*O*-propylnaringenin oxime (**8b**, IC_50_ = 4.59 ± 0.56 µg/mL). The IC_50_ values of the most effective anticancer naringenin derivatives (**8b**, **11b**, **13b**, **16b**) were up to four times lower than the IC_50_ value of the positive control, cisplatin (IC_50_ = 16.73 ± 0.58 µg/mL), and slightly weaker than the cytostatic antibiotic, doxorubicin (IC_50_ = 0.33 ± 0.02 µg/mL). According to the literature, the anticancer activity of flavonoids is the effect of their wide biological properties, hence, it is not yet precisely described. It is explained as the outcome of their antioxidant activity, possible carcinogens inactivation, angiogenesis inhibition and promoting ‘healthy’ proliferation and apoptosis [6].

Furthermore, the chemical modifications of naringenin presented in this paper may possibly increase its stability, and hence, its antioxidant activity in human plasma. It has been reported that the hydroxylated and glycosylated flavonoids have weaker affinities towards serum proteins. In contrast, the incorporation of methoxy groups increases the affinity, and flavonoids remaining in the complexes with proteins are protected from the disadvantageous modifications by the serum enzymes [36,37]. Unfortunately, the stability and—as an effect—the desirable pro-health properties of flavonoids may be diminished by their glycosylation, and these compounds may have a weaker effect on diabetics [36].

## 3. Materials and Methods

### 3.1. Chemicals

Reagents for chemical synthesis—naringenin, 1-iodopropane, 2-iodopropane, 1-iodobutane, and 1-iododecane—were purchased from Sigma-Aldrich Co. (St. Louis, MO, USA); hydroxylamine hydrochloride from LOBA Feinchemie GmbH (Fischamed, Austria); anhydrous sodium acetate, potassium carbonate and *N*,*N*-dimethylformamide (DMF) from Chempur (Piekary Śląskie, Poland). Anhydrous solvents for reactions (acetone and ethanol) were prepared according to standard procedures. All organic solvents were of analytical grade.

### 3.2. Analysis

The thin layer chromatography (TLC) analysis was performed to monitor the progress of reactions. We used silica gel-coated aluminum sheets with a fluorescent indicator (DC-Alufolien, Kieselgel 60 F_254_; Merck, Darmstadt, Germany), which were sprayed with a solution of 1% Ce(SO_4_)_2_ and 2% H_3_[P(Mo_3_O_10_)_4_] in 5% H_2_SO_4_ and heated to visualize synthesized compounds. The mixture of crude products was separated and purified by liquid column chromatography using silica gel (Kieselgel 60, 230–400 mesh, Merck). To determine the structures of the obtained products, ^1^H and ^13^C nuclear magnetic resonance (NMR) spectra were recorded on a Bruker Avance^TM^600 MHz spectrometer (Bruker, Billerica, MA, USA). Samples for NMR analysis were prepared using deuterated solvents: acetone-*d*_6_, chloroform-d, and dimethyl sulfoxide-*d*_6_ (Supplementary Materials, Figures S1–S36). Spectroscopic data of compounds **1a**–**6a**, **14a**–**15a**, **18a**–**19a**, **1b**–**6b**, **14b**–**15b**, and **18b**–**19b** were described in our previous work [19].

To confirm the molar masses of the synthesized derivatives, high-resolution ESI-MS spectra were measured on a Bruker ESI-Q-TOF Maxis Impact Mass Spectrometer (Bruker). The direct infusion of ESI-MS parameters: the mass spectrometer was operated in positive ion mode with the potential between the spray needle and the orifice as 3.5 kV, a nebulizer pressure of 0.4 bar, and a drying gas flow rate of 3.0 L/min at 200 °C. The sample flow rate was 3.0 µL/min. Ionization mass spectra were collected in a range of *m*/*z* 50–1250.

The melting points (uncorrected) were determined with a Boetius apparatus (Jena, Germany).

### 3.3. Synthesis of Mono- (***7a***, ***10a***, ***12a***, ***16a***) and Di-O-alkyl Derivatives of Naringenin (***8a***, ***11a***, ***13a***, ***17a***)

To naringenin (7.35 mmol) dissolved in anhydrous acetone (20 mL), anhydrous potassium carbonate (11.02 mmol) and the corresponding alkyl iodide (36.73 mmol) were added. Reactions were performed with stirring at 40–45 °C for 40–96 h. Then, the organic solvent was evaporated, a saturated solution of sodium chloride (40 mL) was added, and extraction with organic solvent (diethyl ether or ethyl acetate) (3 × 50 mL) was carried out. The collected organic fractions were combined, dried over sodium sulfate, evaporated, and the mixture of crude products was separated by column chromatography.

### 3.4. Synthesis of 5,7,4′-Tri-O-propylnaringenin (***9a***)

5,7,4′-Tri-*O*-propylnaringenin (**9a**) was obtained by reaction of naringenin (3.67 mmol) dissolved in DMF (10 mL) with the addition of anhydrous potassium carbonate (22.04 mmol) and 1-iodopropane (132.80 mmol). The solution was stirred for 20 h at room temperature. Then, 1 M HCl was added until pH 7 was reached. The resultant mixture was extracted with ethyl acetate (3 × 50 mL). Collected organic fractions were combined, dried over sodium sulfate and then evaporated in a vacuum evaporator. The crude product was purified by column chromatography.

*7-O-Propylnaringenin* (**7a**), Yield 61.9% (1.428 g), white powder, mp: 155–157 °C; eluent hexane/dichloromethane/ethyl acetate (*v*/*v*/*v*) 5:1:1, Rf_TLC_ = 0.29; ^1^H-NMR (600 MHz, CDCl_3_): δ 12.01 (s, 1H, OH-5), 7.35–7.31 (m, 2H, AA′BB′, H-2′, H-6′), 6.91–6.86 (m, 2H, AA′BB′, H-3′, H-5′), 6.06 (d, *J* = 2.3 Hz, 1H, H-6), 6.03 (d, *J* = 2.3 Hz, 1H, H-8), 5.35 (dd, *J* = 13.0, 3.0 Hz, 1H, H-2), 5.20 (s, 1H, OH-4′), 3.92 (t, *J* = 6.6 Hz, 2H, -CH_2_-), 3.08 (dd, *J* = 17.1, 13.0 Hz, 1H, H-3_a_), 2.78 (dd, *J* = 17.1, 3.0 Hz, 1H, H-3_b_), 1.79 (h, *J* = 7.4 Hz, 2H, -CH_2_-), 1.01 (t, *J* = 7.4 Hz, 3H, -CH_3_); ^13^C-NMR (150 MHz, CDCl_3_): δ 196.14 (C=O), 167.80, 164.20, 163.01, 156.24, 130.74, 128.10, 115.80, 103.14, 95.71, 94.77, 79.05, 70.16, 43.32, 22.40, 10.51; HRMS (*m*/*z*): [M + H]^+^ calcd. for C_18_H_19_O_5_, 315.1227; found 315.1231.

*7,4′-Di-O-propylnaringenin* (**8a**), Yield 28.3% (0.741 g), white powder, mp: 93–96 °C; eluent hexane/dichloromethane/ethyl acetate (*v*/*v*/*v*) 5:1:1, Rf_TLC_ = 0.71; ^1^H-NMR (600 MHz, CDCl_3_): δ 12.02 (s, 1H, OH-5), 7.39–7.33 (m, 2H, AA′BB′, H-2′, H-6′), 6.97–6.91 (m, 2H, AA′BB′, H-3′, H-5′), 6.05 (d, *J* = 2.3 Hz, 1H, H-6), 6.03 (d, *J* = 2.3 Hz, 1H, H-8), 5.35 (dd, *J* = 13.0, 3.0 Hz, 1H, H-2), 3.96–3.90 (m, 4H, 2x-CH_2_-), 3.09 (dd, *J* = 17.1, 13.0 Hz, 1H, H-3_a_), 2.77 (dd, *J* = 17.1, 3.0 Hz, 1H, H-3_b_), 1.86–1.75 (m, 4H, 2x-CH_2_-), 1.04 (t, *J* = 7.4 Hz, 3H, -CH_3_), 1.01 (t, *J* = 7.4 Hz, 3H, -CH_3_); ^13^C-NMR (150 MHz, CDCl_3_): δ 196.10 (C=O), 167.68, 164.20, 163.02, 159.72, 130.33, 127.81, 114.88, 103.13, 95.63, 94.69, 79.12, 70.10, 69.74, 43.31, 22.66, 22.39, 10.64, 10.50; HRMS (*m*/*z*): [M + H]^+^ calcd. for C_21_H_25_O_5_, 357.1697; found 357.1708.

*5,7,4′-Tri-O-propylnaringenin* (**9a**), Yield 29.9% (0.437 g), white powder, mp: 73–77 °C; eluent hexane/dichloromethane/ethyl acetate (*v*/*v*/*v*) 5:1:1, Rf_TLC_ = 0.52; ^1^H-NMR (600 MHz, CDCl_3_): δ 7.39–7.34 (m, 2H, AA′BB′, H-2′, H-6′), 6.95–6.90 (m, 2H, AA′BB′, H-3′, H-5′), 6.10 (d, *J* = 2.3 Hz, 1H, H-6), 6.07 (d, *J* = 2.3 Hz, 1H, H-8), 5.33 (dd, *J* = 13.3, 2.8 Hz, 1H, H-2), 4.02–3.87 (m, 6H, 3x-CH_2_-), 3.02 (dd, *J* = 16.5, 13.3 Hz, 1H, H-3_a_), 2.72 (dd, *J* = 16.5, 2.8 Hz, 1H, H-3_b_), 1.94–1.87 (m, 2H, -CH_2_-), 1.85–1.76 (m, 4H, 2x-CH_2_-), 1.10 (t, *J* = 7.4 Hz, 3H, -CH_3_), 1.04 (t, *J* = 7.4 Hz, 3H, -CH_3_), 1.02 (t, *J* = 7.4 Hz, 3H, -CH_3_); ^13^C-NMR (150 MHz, CDCl_3_): δ 189.34 (C=O), 165.50, 165.08, 161.92, 159.55, 130.85, 127.78, 114.79, 106.10, 94.25, 93.89, 79.12, 70.57, 69.92, 69.71, 45.70, 22.66, 22.51, 22.46, 10.65, 10.63, 10.55; HRMS (*m*/*z*): [M + H]^+^ calcd. for C_24_H_31_O_5_, 399.2166; found 399.2174.

*7-O-Isopropylnaringenin* (**10a**), Yield 66.4% (1.533 g), yellow powder, mp: 56–60 °C; eluent hexane/dichloromethane/ethyl acetate (*v*/*v*/*v*) 5:1:1, Rf_TLC_ = 0.23; ^1^H-NMR (600 MHz, CDCl_3_): δ 12.00 (s, 1H, OH-5), 7.35–7.29 (m, 2H, AA′BB′, H-2′, H-6′), 6.91–6.85 (m, 2H, AA′BB′, H-3′, H-5′), 6.04 (d, *J* = 2.3 Hz, 1H, H-6), 6.01 (d, *J* = 2.3 Hz, 1H, H-8), 5.48 (s, 1H, OH-4′), 5.34 (dd, *J* = 13.1, 3.0 Hz, 1H, H-2), 4.59–4.52 (m, 1H, -CH-), 3.08 (dd, *J* = 17.1, 13.1 Hz, 1H, H-3_a_), 2.77 (dd, *J* = 17.1, 3.0 Hz, 1H, H-3_b_), 1.35–1.32 (m, 6H, 2x-CH_3_); ^13^C-NMR (150 MHz, CDCl_3_): δ 196.09 (C=O), 166.80, 164.20, 163.10, 156.35, 130.64, 128.11, 115.82, 102.97, 96.38, 95.39, 79.06, 70.82, 43.29, 22.06, 22.02; HRMS (*m*/*z*): [M + H]^+^ calcd. for C_18_H_19_O_5_, 315.1227; found 315.1239.

*7,4′-Di-O-isopropylnaringenin* (**11a**), Yield 28.1% (0.736 g), yellow oil; eluent hexane/dichloromethane/ethyl acetate (*v*/*v*/*v*) 5:1:1, Rf_TLC_ = 0.70; ^1^H-NMR (600 MHz, CDCl_3_): δ 12.01 (s, 1H, OH-5), 7.39–7.32 (m, 2H, AA′BB′, H-2′, H-6′), 6.96–6.90 (m, 2H, AA′BB′, H-3′, H-5′), 6.03 (d, *J* = 2.3 Hz, 1H, H-6), 6.00 (d, *J* = 2.3 Hz, 1H, H-8), 5.34 (dd, *J* = 13.1, 2.9 Hz, 1H, H-2), 4.61–4.51 (m, 2H, 2x-CH-), 3.09 (dd, *J* = 17.1, 13.1 Hz, 1H, H-3_a_), 2.77 (dd, *J* = 17.1, 2.9 Hz, 1H, H-3_b_), 1.36–1.32 (m, 12H, 4x-CH_3_); ^13^C-NMR (150 MHz, CDCl_3_): δ 196.02 (C=O), 166.67, 164.23, 163.11, 158.52, 130.25, 127.90, 116.10, 102.97, 96.32, 95.30, 79.16, 70.73, 70.09, 43.31, 22.14, 22.13, 22.06, 22.02; HRMS (*m*/*z*): [M + H]^+^ calcd. for C_21_H_25_O_5_, 357.1697; found 357.1706.

*7-O-Butylnaringenin* (**12a**), Yield 78.7% (1.898 g), white powder, mp: 92–94 °C (lit. [38] 145–146 °C); eluent hexane/dichloromethane/ethyl acetate (*v*/*v*/*v*) 5:1:1, Rf_TLC_ = 0.27; ^1^H-NMR (600 MHz, CDCl_3_): δ 12.01 (s, 1H, OH-5), 7.34–7.29 (m, 2H, AA′BB′, H-2′, H-6′), 6.90–6.86 (m, 2H, AA′BB′, H-3′, H-5′), 6.06 (d, *J* = 2.3 Hz, 1H, H-6), 6.03 (d, *J* = 2.3 Hz, 1H, H-8), 5.68 (s, 1H, OH-4′), 5.34 (dd, *J* = 13.0, 3.0 Hz, 1H, H-2), 3.96 (t, *J* = 6.5 Hz, 2H, -CH_2_-), 3.08 (dd, *J* = 17.2, 13.0 Hz, 1H, H-3_a_), 2.78 (dd, *J* = 17.2, 3.0 Hz, 1H, H-3_b_), 1.77–1.71 (m, 2H, -CH_2_-), 1.50–1.41 (m, 2H, -CH_2_-), 0.96 (t, *J* = 7.4 Hz, 3H, -CH_3_); ^13^C-NMR (150 MHz, CDCl_3_): δ 196.24 (C=O), 167.84, 164.15, 163.03, 156.44, 130.51, 128.07, 115.81, 103.11, 95.70, 94.77, 79.06, 68.41, 43.25, 31.02, 19.23, 13.87; HRMS (*m*/*z*): [M + H]^+^ calcd. for C_19_H_21_O_5_, 329.1384; found 329.1398.

*7,4′-Di-O-butylnaringenin* (**13a**), Yield 16.5% (0.466 g), pale yellow powder, mp: 72–75 °C; eluent hexane/dichloromethane/ethyl acetate (*v*/*v*/*v*) 5:1:1, Rf_TLC_ = 0.75; ^1^H-NMR (600 MHz, CDCl_3_): δ 12.02 (s, 1H, OH-5), 7.39–7.31 (m, 2H, AA′BB′, H-2′, H-6′), 6.97–6.91 (m, 2H, AA′BB′, H-3′, H-5′), 6.05 (d, *J* = 2.3 Hz, 1H, H-6), 6.03 (d, *J* = 2.3 Hz, 1H, H-8), 5.35 (dd, *J* = 13.0, 3.0 Hz, 1H, H-2), 3.98 (t, *J* = 6.5 Hz, 2H, -CH_2_-), 3.96 (t, *J* = 6.5 Hz, 2H, -CH_2_-), 3.09 (dd, *J* = 17.1, 13.0 Hz, 1H, H-3_a_), 2.78 (dd, *J* = 17.1, 3.0 Hz, 1H, H-3_b_), 1.82–1.71 (m, 4H, 2x-CH_2_-), 1.55–1.41 (m, 4H, 2x-CH_2_-), 0.98 (t, *J* = 7.4 Hz, 3H, -CH_3_), 0.96 (t, *J* = 7.4 Hz, 3H, -CH_3_); ^13^C-NMR (150 MHz, CDCl_3_): δ 196.09 (C=O), 167.72, 164.21, 163.03, 159.75, 130.33, 127.81, 114.89, 103.14, 95.65, 94.70, 79.14, 68.37, 67.95, 43.33, 31.39, 31.06, 19.37, 19.25, 13.97, 13.88; HRMS (*m*/*z*): [M + H]^+^ calcd. for C_23_H_29_O_5_, 385.2010; found 385.2018.

*7-O-Decylnaringenin* (**16a**), Yield 70.8% (2.147 g), white powder, mp: 104–105 °C (lit. [38] 106–107 °C); eluent hexane/dichloromethane/ethyl acetate (*v*/*v*/*v*) 10:1:1, Rf_TLC_ = 0.14; ^1^H-NMR (600 MHz, CDCl_3_): δ 12.01 (s, 1H, OH-5), 7.35–7.30 (m, 2H, AA′BB′, H-2′, H-6′), 6.91–6.86 (m, 2H, AA′BB′, H-3′, H-5′), 6.06 (d, *J* = 2.2 Hz, 1H, H-6), 6.03 (d, *J* = 2.2 Hz, 1H, H-8), 5.40 (s, 1H, OH-4′), 5.34 (dd, *J* = 13.0, 3.0 Hz, 1H, H-2), 3.95 (t, *J* = 6.6 Hz, 2H, -CH_2_-), 3.08 (dd, *J* = 17.1, 13.0 Hz, 1H, H-3_a_), 2.78 (dd, *J* = 17.2, 3.0 Hz, 1H, H-3_b_), 1.78–1.73 (m, 2H, -CH_2_-), 1.44–1.38 (m, 2H, -CH_2_-), 1.34–1.24 (m, 12H, 6x-CH_2_-), 0.88 (t, *J* = 6.9 Hz, 3H, -CH_3_); ^13^C-NMR (150 MHz, CDCl_3_): δ 196.18 (C=O), 167.84, 164.18, 163.02, 156.29, 130.67, 128.09, 115.80, 103.12, 95.72, 94.78, 79.05, 68.74, 43.29, 32.02, 29.66, 29.44, 29.42, 29.01, 26.02, 22.82, 14.26; HRMS (*m*/*z*): [M + H]^+^ calcd. for C_25_H_33_O_5_, 413.2323; found 413.2333.

*7,4′-Di-O-decylnaringenin* (**17a**), Yield 13.6% (0.552 g), pale yellow powder, mp: 48–50 °C; eluent hexane/dichloromethane/ethyl acetate (*v*/*v*/*v*) 10:1:1, Rf_TLC_ = 0.73; ^1^H-NMR (600 MHz, CDCl_3_): δ 12.02 (s, 1H, OH-5), 7.39–7.33 (m, 2H, AA′BB′, H-2′, H-6′), 6.96–6.91 (m, 2H, AA′BB′, H-3′, H-5′), 6.05 (d, *J* = 2.3 Hz, 1H, H-6), 6.02 (d, *J* = 2.3 Hz, 1H, H-8), 5.35 (dd, *J* = 13.0, 3.0 Hz, 1H, H-2), 3.97 (t, *J* = 6.6 Hz, 2H, -CH_2_-), 3.95 (t, *J* = 6.6 Hz, 2H, -CH_2_-), 3.09 (dd, *J* = 17.1, 13.0 Hz, 1H, H-3_a_), 2.78 (dd, *J* = 17.1, 3.0 Hz, 1H, H-3_b_), 1.83–1.72 (m, 4H, 2x-CH_2_-), 1.48–1.39 (m, 4H, 2x-CH_2_-), 1.36–1.25 (m, 24H, 12x-CH_2_-), 0.89 (t, *J* = 6.9 Hz, 3H, -CH_3_), 0.88 (t, *J* = 6.9 Hz, 3H, -CH_3_); ^13^C-NMR (150 MHz, CDCl_3_): δ 196.10 (C=O), 167.72, 164.22, 163.03, 159.75, 130.32, 127.82, 114.89, 103.14, 95.66, 94.70, 79.15, 68.69, 68.27, 43.34, 32.04, 32.03, 29.72, 29.70, 29.67, 29.53, 29.46, 29.45, 29.43, 29.35, 29.03, 26.17, 26.03, 22.83, 22.82, 14.27, 14.26; HRMS (*m*/*z*): [M + H]^+^ calcd. for C_35_H_53_O_5_, 553.3888; found 553.3884.

### 3.5. Synthesis of Oximes (***7b***–***13b***, ***16b***–***17b***)

To a solution of an *O*-alkyl derivative of naringenin (0.48 mmol) in anhydrous ethanol (10 mL), hydroxylamine hydrochloride (0.72 mmol) and anhydrous sodium acetate (0.72 mmol) were added. The reaction was carried out with a magnetic stirrer at 40–50 °C. After completing the reaction, the mixture was poured into ice water, and the precipitated crystals were collected. The crude product was purified by column chromatography.

*7-O-Propylnaringenin oxime* (**7b**), Yield 87.7% (0.132 g), white powder, mp: 207–210 °C; eluent chloroform/methanol (*v*/*v*) 96:4, Rf_TLC_ = 0.50; ^1^H-NMR (600 MHz, (CD_3_)_2_CO): δ 11.02 (s, 1H, NOH), 10.41 (s, 1H, OH-5), 8.51 (s, 1H, OH-4′), 7.40–7.36 (m, 2H, AA′BB′, H-2′, H-6′), 6.91–6.87 (m, 2H, AA′BB′, H-3′, H-5′), 6.05 (d, *J* = 2.4 Hz, 1H, H-6), 6.03 (d, *J* = 2.4 Hz, 1H, H-8), 5.07 (dd, *J* = 12.0, 3.1 Hz, 1H, H-2), 3.92 (t, *J* = 6.5 Hz, 2H, -CH_2_-), 3.46 (dd, *J* = 17.1, 3.1 Hz, 1H, H-3_a_), 2.79 (dd, *J* = 17.1, 12.0 Hz, 1H, H-3_b_), 1.79–1.71 (m, 2H, -CH_2_-), 1.00 (t, *J* = 7.4 Hz, 3H, -CH_3_); ^13^C-NMR (150 MHz, (CD_3_)_2_CO): δ 162.92, 160.61, 159.39, 158.45, 154.83 (C=NOH), 131.73, 128.78, 116.11, 99.13, 96.56, 95.09, 77.36, 70.11, 30.28, 23.11, 10.68; HRMS (*m*/*z*): [M + H]^+^ calcd. for C_18_H_20_NO_5_, 330.1336; found 330.1334.

*7,4′-Di-O-propylnaringenin oxime* (**8b**), Yield 90.1% (0.153 g), white powder, mp: 113–115 °C; eluent chloroform/methanol (*v*/*v*) 99:1, Rf_TLC_ = 0.66; ^1^H-NMR (600 MHz, (CD_3_)_2_CO): δ 11.02 (s, 1H, NOH), 10.43 (s, 1H, OH-5), 7.49–7.42 (m, 2H, AA′BB′, H-2′, H-6′), 7.01–6.95 (m, 2H, AA′BB′, H-3′, H-5′), 6.05 (d, *J* = 2.4 Hz, 1H, H-6), 6.04 (d, *J* = 2.4 Hz, 1H, H-8), 5.11 (dd, *J* = 11.9, 3.2 Hz, 1H, H-2), 3.97 (t, *J* = 6.5 Hz, 2H, -CH_2_-), 3.92 (t, *J* = 6.5 Hz, 2H, -CH_2_-), 3.47 (dd, *J* = 17.1, 3.2 Hz, 1H, H-3_a_), 2.80 (dd, *J* = 17.1, 11.9 Hz, 1H, H-3_b_), 1.84–1.77 (m, 2H, -CH_2_-), 1.77–1.70 (m, 2H, -CH_2_-), 1.03 (t, *J* = 7.4 Hz, 3H, -CH_3_), 1.00 (t, *J* = 7.4 Hz, 3H, -CH_3_); ^13^C-NMR (150 MHz, (CD_3_)_2_CO): δ 162.93, 160.62, 160.21, 159.31, 154.72 (C=NOH), 132.72, 128.65, 115.25, 99.14, 96.59, 95.12, 77.20, 70.12, 70.07, 30.26, 23.25, 23.12, 10.76, 10.69; HRMS (*m*/*z*): [M + H]^+^ calcd. for C_21_H_26_NO_5_, 372.1805; found 372.1810.

*5,7,4′-Tri-O-propylnaringenin oxime* (**9b**), Yield 93.6% (0.187 g), white powder, mp: 166–169 °C; eluent chloroform/methanol (*v*/*v*) 98:2, Rf_TLC_ = 0.21; ^1^H-NMR (600 MHz, (CD_3_)_2_SO): δ 10.81 (s, 1H, NOH), 7.40–7.35 (m, 2H, AA′BB′, H-2′, H-6′), 6.96–6.91 (m, 2H, AA′BB′, H-3′, H-5′), 6.18 (d, *J* = 2.4 Hz, 1H, H-6), 6.12 (d, *J* = 2.4 Hz, 1H, H-8), 5.00 (dd, *J* = 11.6, 3.3 Hz, 1H, H-2), 3.96–3.90 (m, 4H, 2x-CH_2_-), 3.89 (t, *J* = 6.6 Hz, 2H, -CH_2_-), 3.31 (dd, *J* = 16.8, 3.3 Hz, 1H, H-3_a_), 2.68 (dd, *J* = 16.8, 11.6 Hz, 1H, H-3_b_), 1.75–1.66 (m, 6H, 3x-CH_2_-), 1.00 (t, *J* = 7.4 Hz, 3H, -CH_3_), 0.97 (t, *J* = 7.4 Hz, 3H, -CH_3_), 0.95 (t, *J* = 7.4 Hz, 3H, -CH_3_); ^13^C-NMR (150 MHz, (CD_3_)_2_SO): δ 160.15, 158.64, 158.57, 158.52, 147.77 (C=NOH), 131.92, 127.78, 114.25, 102.20, 94.65, 94.43, 75.98, 69.71, 69.01, 68.94, 30.28, 22.04, 22.01, 21.95, 10.68, 10.41, 10.39; HRMS (*m*/*z*): [M + H]^+^ calcd. for C_24_H_32_NO_5_, 414.2275; found 414.2281.

*7-O-Isopropylnaringenin oxime* (**10b**), Yield 77.5% (0.154 g), white powder, mp: 200–204 °C; eluent chloroform/methanol (*v*/*v*) 98:2, Rf_TLC_ = 0.11; ^1^H-NMR (600 MHz, (CD_3_)_2_CO): δ 10.99 (s, 1H, NOH), 10.37 (s, 1H, OH-5), 8.48 (s, 1H, OH-4′), 7.41–7.36 (m, 2H, AA′BB′, H-2′, H-6′), 6.92–6.86 (m, 2H, AA′BB′, H-3′, H-5′), 6.03 (d, *J* = 2.4 Hz, 1H, H-6), 6.01 (d, *J* = 2.4 Hz, 1H, H-8), 5.07 (dd, *J* = 12.0, 3.1 Hz, 1H, H-2), 4.63–4.56 (m, 1H, -CH-), 3.46 (dd, *J* = 17.1, 3.1 Hz, 1H, H-3_a_), 2.78 (dd, *J* = 17.1, 12.0 Hz, 1H, H-3_b_), 1.30–1.26 (m, 6H, 2x-CH_3_);^13^C-NMR (150 MHz, (CD_3_)_2_CO): δ 161.75, 160.64, 159.45, 158.43, 154.85 (C=NOH), 131.75, 128.80, 116.11, 98.98, 97.45, 95.97, 77.35, 70.35, 30.29, 22.26, 22.21; HRMS (*m*/*z*): [M + H]^+^ calcd. for C_18_H_20_NO_5_, 330.1336; found 330.1354.

*7,4′-Di-O-isopropylnaringenin oxime* (**11b**), Yield 87.4% (0.137 g), white powder, mp: 95–98 °C; eluent chloroform/methanol (*v*/*v*) 99:1, Rf_TLC_ = 0.59; ^1^H-NMR (600 MHz, (CD_3_)_2_CO): δ 10.99 (s, 1H, NOH), 10.39 (s, 1H, OH-5), 7.47–7.43 (m, 2H, AA′BB′, H-2′, H-6′), 6.98–6.95 (m, 2H, AA′BB′, H-3′, H-5′), 6.03 (d, *J* = 2.4 Hz, 1H, H-6), 6.02 (d, *J* = 2.4 Hz, 1H, H-8), 5.10 (dd, *J* = 12.0, 3.2 Hz, 1H, H-2), 4.69–4.63 (m, 1H, -CH-), 4.63–4.56 (m, 1H, -CH-), 3.47 (dd, *J* = 17.1, 3.2 Hz, 1H, H-3_a_), 2.80 (dd, *J* = 17.1, 12.0 Hz, 1H, H-3_b_), 1.31 (d, *J* = 6.0 Hz, 6H, 2x-CH_3_), 1.29–1.27 (m, 6H, 2x-CH_3_); ^13^C-NMR (150 MHz, (CD_3_)_2_CO): δ 161.77, 160.66, 159.39, 159.01, 154.76 (C=NOH), 132.64, 128.73, 116.48, 99.00, 97.48, 96.00, 77.24, 70.37, 70.29, 30.27, 22.27, 22.22; HRMS (*m*/*z*): [M + H]^+^ calcd. for C_21_H_26_NO_5_, 372.1805; found 372.1819.

*7-O-Butylnaringenin oxime* (**12b**), Yield 82.8% (0.433 g), white powder, mp: 204–207 °C; eluent chloroform/methanol (*v*/*v*) 98:2, Rf_TLC_ = 0.13; ^1^H-NMR (600 MHz, (CD_3_)_2_CO): δ 11.01 (s, 1H, NOH), 10.35 (s, 1H, OH-5), 8.45 (s, 1H, OH-4′), 7.40–7.36 (m, 2H, AA′BB′, H-2′, H-6′), 6.92–6.86 (m, 2H, AA′BB′, H-3′, H-5′), 6.05 (d, *J* = 2.4 Hz, 1H, H-6), 6.03 (d, *J* = 2.4 Hz, 1H, H-8), 5.07 (dd, *J* = 12.0, 3.1 Hz, 1H, H-2), 3.97 (t, *J* = 6.5 Hz, 2H, -CH_2_-), 3.46 (dd, *J* = 17.1, 3.1 Hz, 1H, H-3_a_), 2.79 (dd, *J* = 17.1, 12.0 Hz, 1H, H-3_b_), 1.76–1.68 (m, 2H, -CH_2_-), 1.51–1.43 (m, 2H, -CH_2_-), 0.95 (t, *J* = 7.4 Hz, 3H, -CH_3_); ^13^C-NMR (150 MHz, (CD_3_)_2_CO): δ 162.93, 160.60, 159.40, 158.43, 154.86 (C=NOH), 131.75, 128.79, 116.11, 99.11, 96.56, 95.09, 77.35, 68.34, 31.91, 30.29, 19.83, 14.06; HRMS (*m*/*z*): [M + H]^+^ calcd. for C_19_H_22_NO_5_, 344.1492; found 344.1506.

*7,4′-Di-O-butylnaringenin oxime* (**13b**), Yield 86.6% (0.180 g), white powder, mp: 82–84 °C; eluent chloroform/methanol (*v*/*v*) 99:0.25, Rf_TLC_ = 0.45; ^1^H-NMR (600 MHz, (CD_3_)_2_CO): δ 11.01 (s, 1H, NOH), 10.38 (s, 1H, OH-5), 7.48–7.43 (m, 2H, AA′BB′, H-2′, H-6′), 7.00–6.95 (m, 2H, AA′BB′, H-3′, H-5′), 6.05 (d, *J* = 2.4 Hz, 1H, H-6), 6.04 (d, *J* = 2.4 Hz, 1H, H-8), 5.10 (dd, *J* = 11.9, 3.1 Hz, 1H, H-2), 4.02 (t, *J* = 6.5 Hz, 2H, -CH_2_-), 3.96 (t, *J* = 6.5 Hz, 2H, -CH_2_-), 3.47 (dd, *J* = 17.1, 3.1 Hz, 1H, H-3_a_), 2.80 (dd, *J* = 17.1, 11.9 Hz, 1H, H-3_b_), 1.80–1.68 (m, 4H, 2x-CH_2_-), 1.54–1.43 (m, 4H, 2x-CH_2_-), 0.97 (t, *J* = 7.4 Hz, 3H, -CH_3_), 0.95 (t, *J* = 7.4 Hz, 3H, -CH_3_); ^13^C-NMR (150 MHz, (CD_3_)_2_CO): δ 162.95, 160.62, 160.22, 159.31, 154.75 (C=NOH), 132.70, 128.65, 115.24, 99.13, 96.59, 95.12, 77.21, 68.35, 68.27, 32.07, 31.92, 30.27, 19.89, 19.83, 14.11, 14.07; HRMS (*m*/*z*): [M + H]^+^ calcd. for C_23_H_30_NO_5_, 400.2118; found 400.2127.

*7-O-Decylnaringenin oxime* (**16b**), Yield 87.0% (0.451 g), white powder, mp: 166–168 °C; eluent chloroform/methanol (*v*/*v*) 98:2, Rf_TLC_ = 0.18; ^1^H-NMR (600 MHz, (CD_3_)_2_CO): δ 11.01 (s, 1H, NOH), 10.37 (s, 1H, OH-5), 8.47 (s, 1H, OH-4′), 7.40–7.36 (m, 2H, AA′BB′, H-2′, H-6′), 6.92–6.87 (m, 2H, AA′BB′, H-3′, H-5′), 6.05 (d, *J* = 2.4 Hz, 1H, H-6), 6.03 (d, *J* = 2.4 Hz, 1H, H-8), 5.07 (dd, *J* = 12.0, 3.1 Hz, 1H, H-2), 3.96 (t, *J* = 6.5 Hz, 2H, -CH_2_-), 3.46 (dd, *J* = 17.1, 3.1 Hz, 1H, H-3_a_), 2.79 (dd, *J* = 17.1, 12.0 Hz, 1H, H-3_b_), 1.77–1.71 (m, 2H, -CH_2_-), 1.48–1.42 (m, 2H, -CH_2_-), 1.37–1.26 (m, 12H, 6x-CH_2_-), 0.87 (t, *J* = 7.0 Hz, 3H, -CH_3_); ^13^C-NMR (150 MHz, (CD_3_)_2_CO): δ 162.93, 160.61, 159.40, 158.43, 154.85 (C=NOH), 131.75, 128.78, 116.11, 99.11, 96.58, 95.10, 77.36, 68.64, 32.62, 30.32, 30.30, 30.28, 30.06, 30.04, 26.70, 23.32, 14.36; HRMS (*m*/*z*): [M + H]^+^ calcd. for C_25_H_34_NO_5_, 428.2431; found 428.2449.

*7,4′-Di-O-decylnaringenin oxime* (**17b**), Yield 91.2% (0.187 g), white powder, mp: 75–78 °C; eluent chloroform/methanol (*v*/*v*) 99:0.25, Rf_TLC_ = 0.66; ^1^H-NMR (600 MHz, (CD_3_)_2_CO): δ 11.01 (s, 1H, NOH), 10.39 (s, 1H, OH-5), 7.48–7.42 (m, 2H, AA′BB′, H-2′, H-6′), 7.00–6.95 (m, 2H, AA′BB′, H-3′, H-5′), 6.05 (d, *J* = 2.4 Hz, 1H, H-6), 6.04 (d, *J* = 2.4 Hz, 1H, H-8), 5.10 (dd, *J* = 11.9, 3.1 Hz, 1H, H-2), 4.02 (t, *J* = 6.5 Hz, 2H, -CH_2_-), 3.96 (t, *J* = 6.5 Hz, 2H, -CH_2_-), 3.47 (dd, *J* = 17.1, 3.1 Hz, 1H, H-3_a_), 2.80 (dd, *J* = 17.1, 11.9 Hz, 1H H-3_b_), 1.81–1.71 (m, 4H, 2x-CH_2_-), 1.51–1.42 (m, 4H, 2x-CH_2_-), 1.39–1.28 (m, 24H, 12x-CH_2_-), 0.88 (t, *J* = 7,0 Hz, 3H, -CH_3_), 0.88 (t, *J* = 7,0 Hz, 3H, -CH_3_); ^13^C-NMR (150 MHz, (CD_3_)_2_CO): δ 162.94, 160.62, 160.22, 159.31, 154.74 (C=NOH), 132.70, 128.64, 115.25, 99.13, 96.61, 95.13, 77.21, 68.65, 68.58, 32.63, 30.35, 30.33, 30.31, 30.29, 30.13, 30.07, 30.05, 30.01, 26.78, 26.71, 23.33, 14.37; HRMS (*m*/*z*): [M + H]^+^ calcd. for C_35_H_54_NO_5_, 568.3997; found 568.4001.

### 3.6. Minimal Inhibitory Concentration (MIC) Evaluation

All bacterial strains (*Escherichia coli* ATCC25922, *Bacillus subtilis* ATCC19659 and *Staphylococcus aureus* ATCC11632) were obtained from the collection of the Polish Academy of Sciences (Wrocław, Poland). Antibiotics were purchased from Sigma-Aldrich Co. (St. Louis, MO, USA). Bacteria were maintained on nutrient agar (Biocorp, Poland) slopes and passaged every month. For antibacterial activity assays, the culture samples were prepared according to CLSI protocol [31]. Briefly, bacteria grown on Mueller-Hinton agar (Biocorp) plates overnight were suspended in Mueller-Hinton broth and adjusted to 0.5 McFarland standard. Incubations were prepared in triplicate on 96-well microplates (TPP, Switzerland) in Mueller-Hinton Broth (Biocorp) with series of two-fold dilutions of tested compounds and 10^5^ CFU/mL of initial bacterial cell density. Plates were incubated overnight at 37 °C with gentle shaking in an ELMI DTS-4 SkyLine orbital shaker. The results were read at a wavelength of 650 nm with a Tecan microplate reader coupled with Magellan software. Minimal inhibitory concentration was defined as the lowest concentration of tested compound that completely restricted the growth of microorganism. Assays were repeated for a minimum of 3 times using separate bacterial cultures.

### 3.7. Sulforhodamine B (SRB) Assay

Human colon adenocarcinoma HT-29 cells (ATCC, Manassas, VA, USA) were cultured in α-MEM medium (IIET PAS, Wrocław, Poland) supplemented with 10% fetal bovine serum, 2 mM glutamine, 100 μg/mL penicillin, 100 μg/mL streptomycin, and 0.25 μg/mL amphotericin (all supplements purchased from Gibco, Paisley, UK). HT-29 cells were incubated at 37 °C, 5% CO_2_ in HeraCELL 150i incubator (Thermo Fisher Scientific, Waltham, MA, USA) and passaged using a trypsin/EDTA solution (IIET PAS). The density of cells was determined using a Bürker chamber.

For the SRB assay, cells at a density of 5 × 10^5^/mL were seeded onto 96-well plates (Sarstedt, Germany) and cultured overnight as described above. Next, the growth medium was gently removed and replaced with fresh α-MEM medium, supplemented with the examined compounds. Stock solutions of naringenin derivatives were prepared in DMSO at a concentration of 10 mg/mL and diluted in sterile α-MEM medium for the incubation step. Cells were incubated for 48 h with the tested compounds. At the end of cell treatment, the cultured medium was replaced with fresh α-MEM, and HT-29 cells were fixed with ice-cold 50% trichloroacetic acid for 1 h at 4 °C. The solution was subsequently removed, and fixed cells were washed five times with water and dried. Fixed HT-29 cells were stained using a solution of 0.4% sulforhodamine B (Sigma-Aldrich Co., St. Louis, MO, USA) in 1% acetic acid (50 µL/well) in darkness for 30 min and rinsed 4-times with 1% acetic acid. Finally, the fixed cells were dried, and 150 µL of 10 mM Tris-HCl buffer was added to each well with intense shaking. For the optimal solubilization of the probes, they were incubated for the next 30 minutes and the optical density was measured at 570 nm with a microplate reader (Victor3, Perkin Elmer, Boston, MA, USA). Each experiment was performed in quadruplicate. The final results were reported as the IC_50_ values. Cisplatin (Acros Organics, Waltham) and doxorubicin (Fisher BioReagents, Waltham) were used as the positive controls in the assay.

## 4. Conclusions

In the presented paper, we described an efficient method for synthesizing novel naringenin analogues—seven *O*-alkyl derivatives (**7a**–**11a**, **13a**, **17a**) and nine oximes (**7b**–**13b**, **16b**–**17b**). Most of the obtained compounds were characterized as exhibiting interesting antibacterial and anticancer properties.

A group of 40 compounds—16 newly synthesized and 23 already known derivatives and naringenin—were screened for bacteriostatic properties against Gram-negative and Gram-positive bacteria. The highest inhibitory effect was observed for tested *B. subtilis* and *S. aureus* strains. Their growth was strongly restricted by 7-*O*-alkyl derivatives of naringenin (**10a** and **10b** with MIC = 12.5 µg/mL; **12a** with MIC = 6.25 µg/mL). In general, di- and tri-*O*-alkyl analogues had weaker antibacterial characteristics, with the exception of oxime **11b** (MIC = 6.25 µg/mL for *B. subtilis*; MIC = 12.5 µg/mL for *S. aureus*). In addition, further modification of *O*-alkyl derivatives with the oxime group did not significantly alter their antibacterial properties.

Additionally, anticancer characteristic studies allowed us to find 26 active naringenin analogues among the examined compounds. In contrast to the antimicrobial activity results, the introduction of the oxime moiety instead of the carbonyl group had an important influence on antiproliferative properties. Among the tested compounds, the most potent were described with an IC_50_ below 5 µg/mL. Namely, they were the oximes with *O*-propyl, *O*-isopropyl, and *O*-butyl chains attached at the C-7 and C-4′ positions (**8b**, **11b**, **13b**) as well as 7-*O*-decylnaringenin oxime (**16b**). Derivatives with long *O*-alkyl groups at the C-7 position (**16a**–**b**, **18a**–**b**) were also described as affective against the examined cancer cell line, simultaneously exhibiting negligible antibacterial activity. The incorporation of the additional *O*-alkyl group at the C-4′ position (**17a**–**b**, **19a**–**b**) diminished both the antiproliferative and bacteriostatic activities of obtained derivatives.

Considering all performed biological studies, novel oximes of 7-*O*-isopropylnaringenin (**10b**) and 7,4′-di-*O*-isopropylnaringenin (**11b**) were the most active compounds, both showing antibacterial and anticancer properties.

## Figures and Tables

**Table 1:**
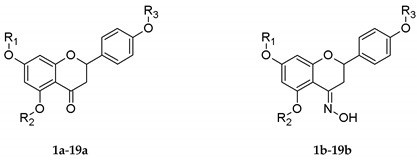
Structures of *O*-alkyl derivatives of naringenin and their oximes.

No.	R_1_	R_2_	R_3_
**1a**, **1b**	Me	H	H
**2a**, **2b**	Me	H	Me
**3a**, **3b**	Me	Me	Me
**4a**, **4b**	Et	H	H
**5a**, **5b**	Et	H	Et
**6a**, **6b**	Et	Et	Et
**7a**^★^, **7b**^★^	*n*-Pr	H	H
**8a**^★^, **8b**^★^	*n*-Pr	H	*n*-Pr
**9a**^★^, **9b**^★^	*n*-Pr	*n*-Pr	*n*-Pr
**10a**^★^, **10b**^★^	*i*-Pr	H	H
**11a**^★^, **11b**^★^	*i*-Pr	H	*i*-Pr
**12a**, **12b**^★^	Bu	H	H
**13a**^★^, **13b**^★^	Bu	H	Bu
**14a**, **14b**	Pe	H	H
**15a**, **15b**	Pe	H	Pe
**16a**, **16b**^★^	De	H	H
**17a**^★^, **17b**^★^	De	H	De
**18a**, **18b**	Dod	H	H
**19a**, **19b**	Dod	H	Dod

^★^ novel derivatives of naringenin. Me—methyl, Et—ethyl, *n*-Pr—propyl, *i*-Pr—isopropyl, Bu—butyl, Pe—pentyl, De—decyl, Dod—dodecyl group.

**Table 2:** Bacteriostatic activity of *O*-alkyl derivatives of naringenin and their oximes calculated as the minimal inhibitory concentration values (µg/mL) (according to the CLSI protocol [31]).

No.	Minimal Inhibitory Concentration (µg/mL)			No.	Minimal Inhibitory Concentration (µg/mL)		
	*Escherichia coli* ATCC25922	*Bacillus subtilis* ATCC19659	*Staphylococcus aureus* ATCC11632		*Escherichia coli* ATCC25922	*Bacillus subtilis* ATCC19659	*Staphylococcus aureus* ATCC11632
**NG**	>400	200	200	**NG-OX**	>400	100	100
**1a**	400	>400	>400	**1b**	400	50	50
**2a**	200	50	50	**2b**	400	100	200
**3a**	400	>400	200	**3b**	>400	>400	400
**4a**	>400	25	25	**4b**	400	25	25
**5a**	400	100	100	**5b**	400	>400	100
**6a**	400	50	100	**6b**	400	>400	100
**7a** ^★^	400	200	100	**7b** ^★^	>400	>400	100
**8a** ^★^	400	>400	>400	**8b** ^★^	400	400	400
**9a** ^★^	>400	>400	>400	**9b** ^★^	>400	>400	>400
**10a** ^★^	>400	12.5	12.5	**10b** ^★^	>400	12.5	12.5
**11a** ^★^	400	>400	>400	**11b** ^★^	400	6.25	12.5
**12a**	400	6.25	6.25	**12b** ^★^	200	50	50
**13a** ^★^	>400	>400	>400	**13b** ^★^	200	100	>400
**14a**	200	50	25	**14b**	200	50	25
**15a**	400	>400	200	**15b**	200	>400	>400
**16a**	>400	100	100	**16b** ^★^	>400	400	200
**17a** ^★^	>400	>400	>400	**17b** ^★^	>400	400	>400
**18a**	400	>400	>400	**18b**	400	>400	>400
**19a**	>400	>400	>400	**19b**	400	200	200
**Gentamicin**					1.5	1.0	1.5
**Nalidixic acid**					6.25	25	50
**Novobiocin**					400	1.0	0.5

^★^ novel derivatives of naringenin.

**Table 3:** Anticancer activity of the *O*-alkyl derivatives of naringenin and their oximes.

No.	HT-29 Cell Line IC_50_ (μg/mL)	No.	HT-29 Cell Line IC_50_ (μg/mL)
**NG**	38.93 ± 13.51	**NG-OX**	29.44 ± 3.16
**1a**	24.98 ± 3.95	**1b**	13.13 ± 1.02
**2a**	>100	**2b**	11.45 ± 0.34
**3a**	20.84 ± 2.05	**3b**	>100
**4a**	14.82 ± 1.25	**4b**	13.75 ± 2.09
**5a**	>100	**5b**	7.65 ± 1.23
**6a**	>100	**6b**	>100
**7a** ^★^	11.99 ± 0.58	**7b** ^★^	9.11 ± 1.34
**8a** ^★^	>100	**8b** ^★^	4.59 ± 0.56
**9a** ^★^	31.77 ± 6.00	**9b** ^★^	>100
**10a** ^★^	10.41 ± 2.14	**10b** ^★^	7.26 ± 0.31
**11a** ^★^	9.81 ± 0.72	**11b** ^★^	4.89 ± 0.56
**12a**	9.71 ± 1.28	**12b** ^★^	6.22 ± 0.30
**13a** ^★^	>100	**13b** ^★^	3.32 ± 0.29
**14a**	13.23 ± 0.61	**14b**	7.00 ± 0.48
**15a**	>100	**15b**	5.89 ± 1.29
**16a**	8.35 ± 0.45	**16b** ^★^	3.63 ± 0.47
**17a** ^★^	>100	**17b** ^★^	>100
**18a**	22.16 ± 4.33	**18b**	7.46 ± 1.21
**19a**	>100	**19b**	>100
**Cisplatin**			16.73 ± 0.58
**Doxorubicin**			0.33 ± 0.02

^★^ novel derivatives of naringenin.

## Supplementary Materials

The supplementary files are available online.
