# Supplementary material for: Novel O-alkyl Derivatives of Naringenin and Their Oximes with Antimicrobial and Anticancer Activity
Source: Molecules. 2019 Feb 14;24(4):679. doi: 10.3390/molecules24040679 (PMC6413393; doi:10.3390/molecules24040679)
Supplement: Supplementary file 1 [file molecules-24-00679-s001.pdf]

Supplementary Information

Novel *O*-alkyl derivatives of naringenin and their oximes with antimicrobial and anticancer activity

Joanna Kozłowska [1,*], Ewa Grela [2], Dagmara Baczyńska [3], Agnieszka Grabowiecka [2], Mirosław Anioł [1]

[1] Department of Chemistry, Faculty of Biotechnology and Food Science, Wrocław University of Environmental and Life Sciences, Norwida 25, 50-375 Wrocław,

[2] Department of Bioorganic Chemistry, Faculty of Chemistry, Wrocław University of Technology, Wybrzeże Wyspiańskiego 27, 50-370 Wrocław, Poland

[3] Department of Molecular and Cellular Biology, Faculty of Pharmacy with Division of Laboratory Diagnostics, Wroclaw Medical University, Borowska 211A, 50-556 Wrocław, Poland

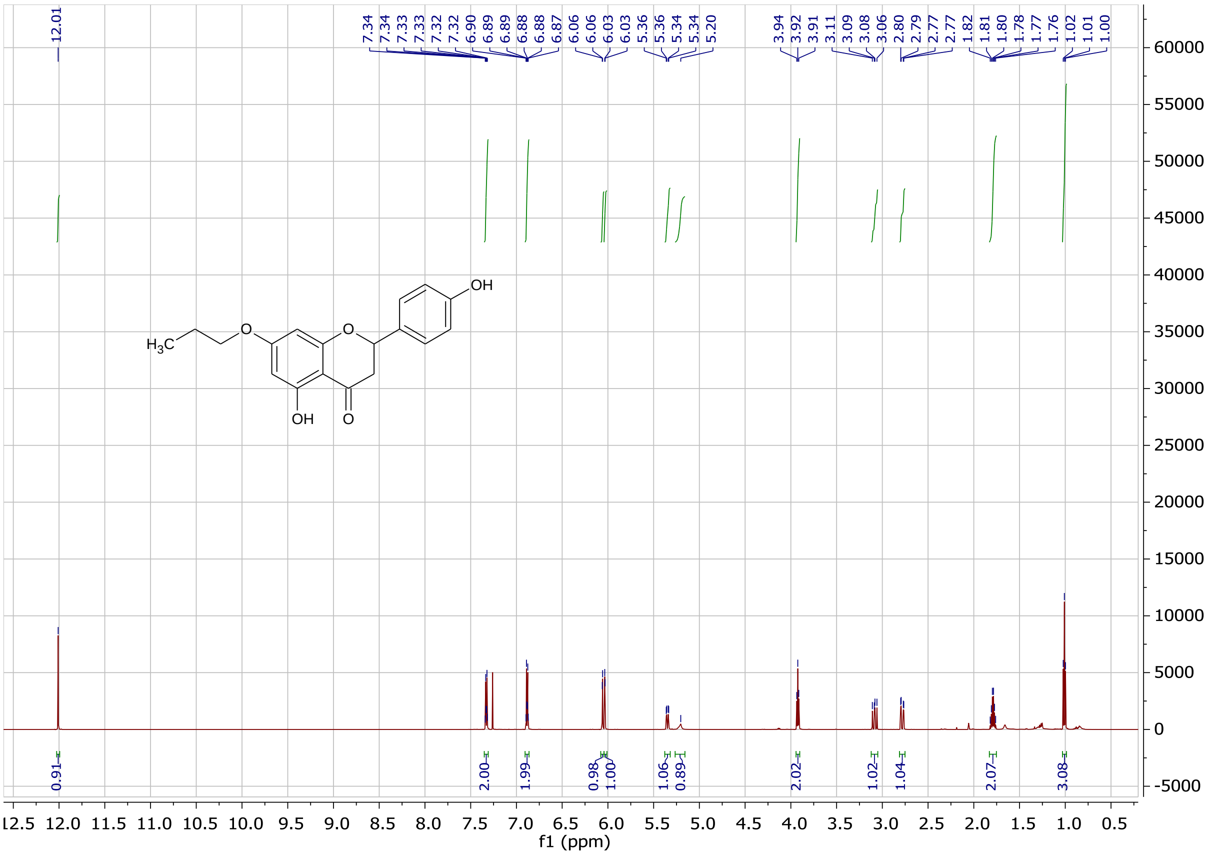

Fig S1. ^{1}H-NMR (600 MHz, $CDCl_3$) spectrum of 7-*O*-Propylnaringenin (**7a**)

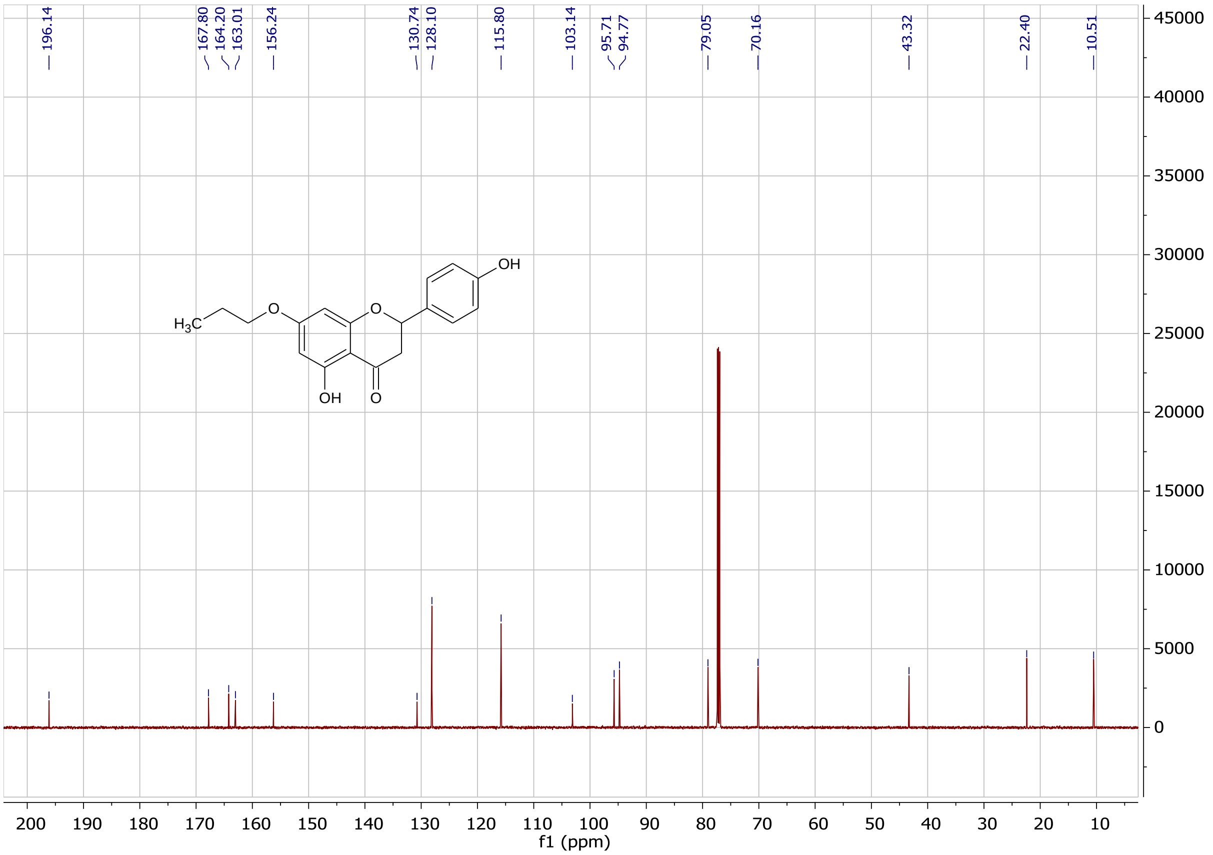

Fig S2. ^{13}C-NMR (150 MHz, $CDCl_3$) spectrum of 7-*O*-Propylnaringenin (**7a**)

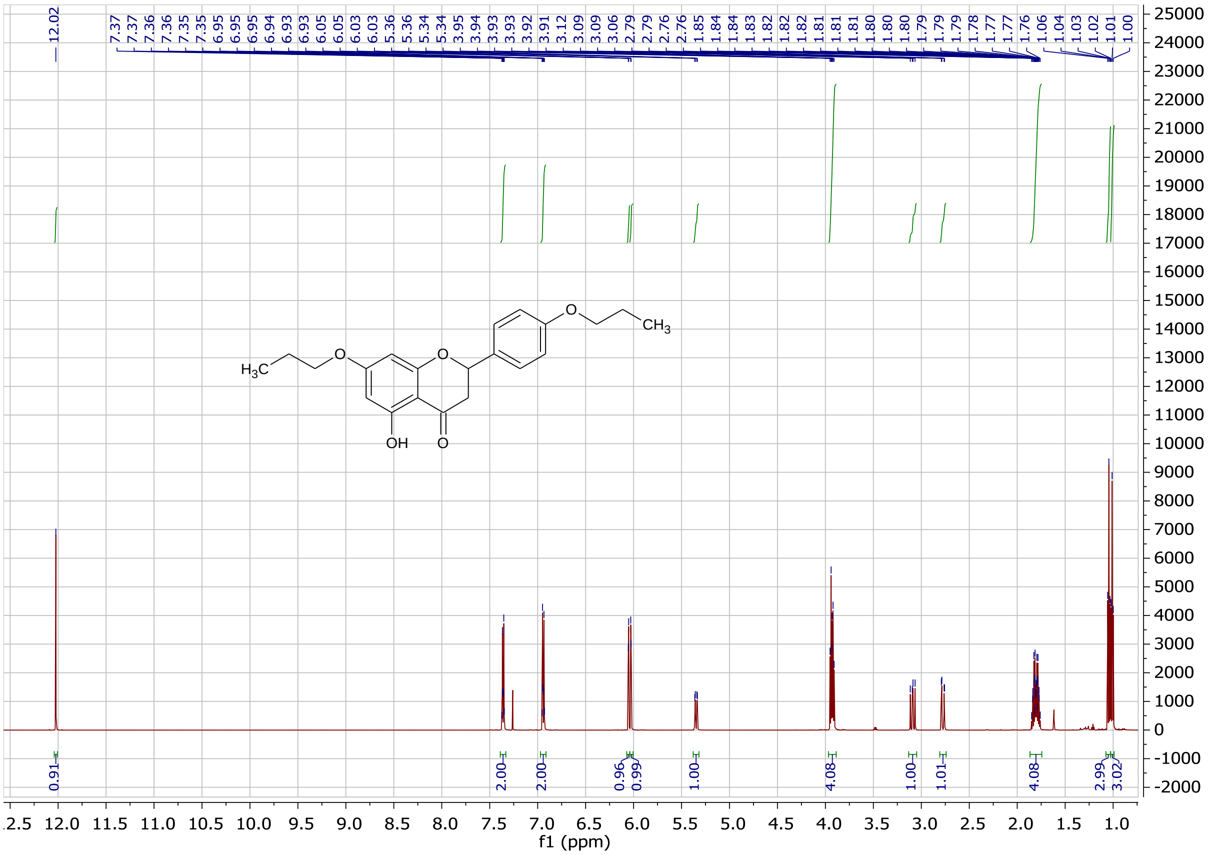

Fig S3. ^{1}H-NMR (600 MHz, $CDCl_3$) spectrum of 7,4′-Di-*O*-propylnaringenin (**8a**)

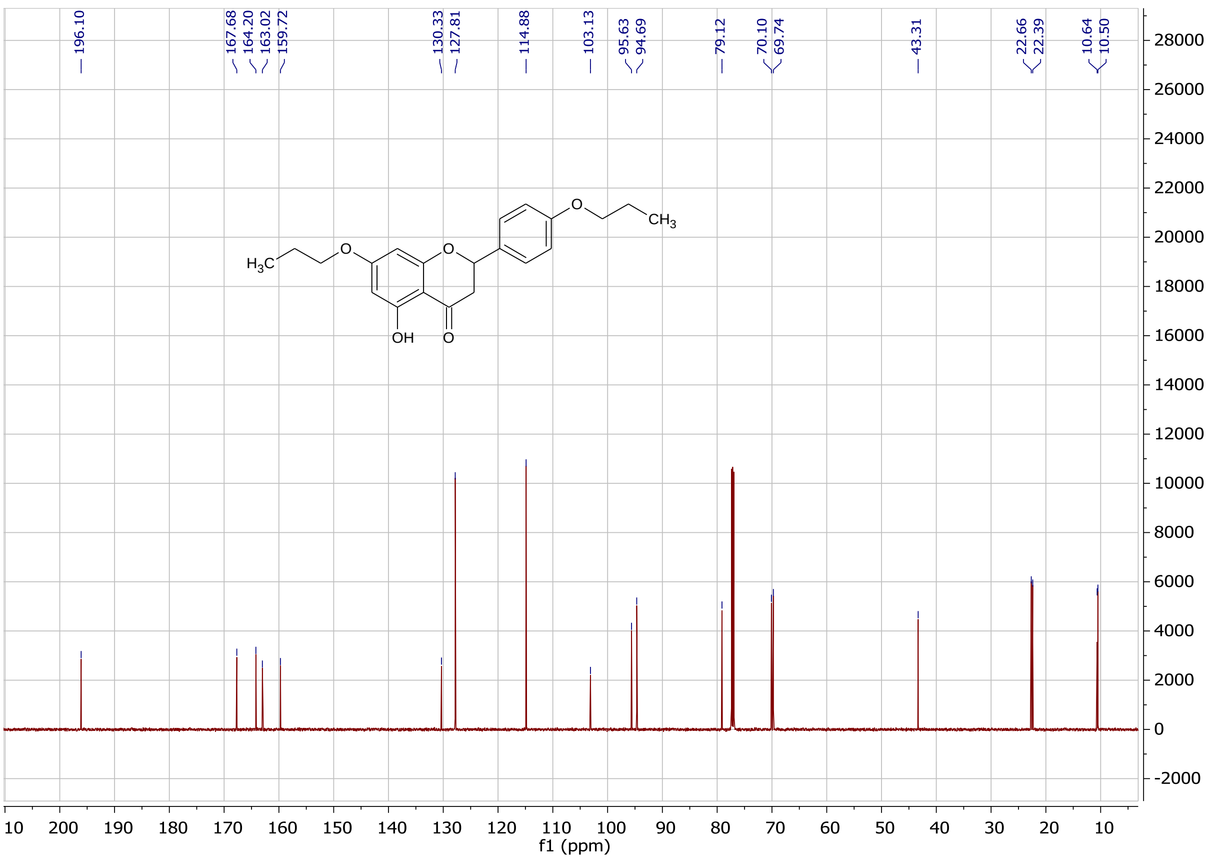

Fig S4. ^{13}C-NMR (150 MHz, CDCl3) spectrum of 7,4′-Di-*O*-propylnaringenin (**8a**)

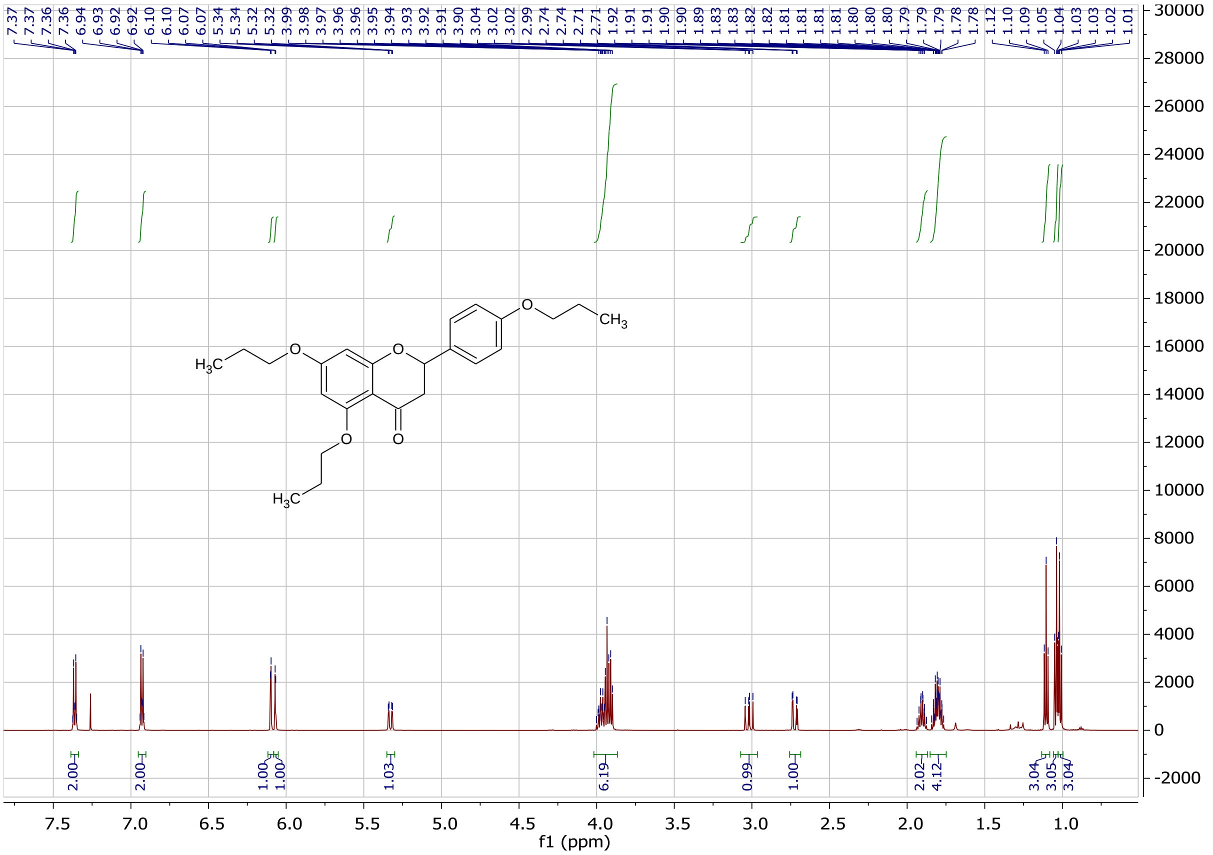

Fig S5. ^{1}H-NMR (600 MHz, CDCl3) spectrum of 5,7,4′-Tri-*O*-propylnaringenin (**9a**)

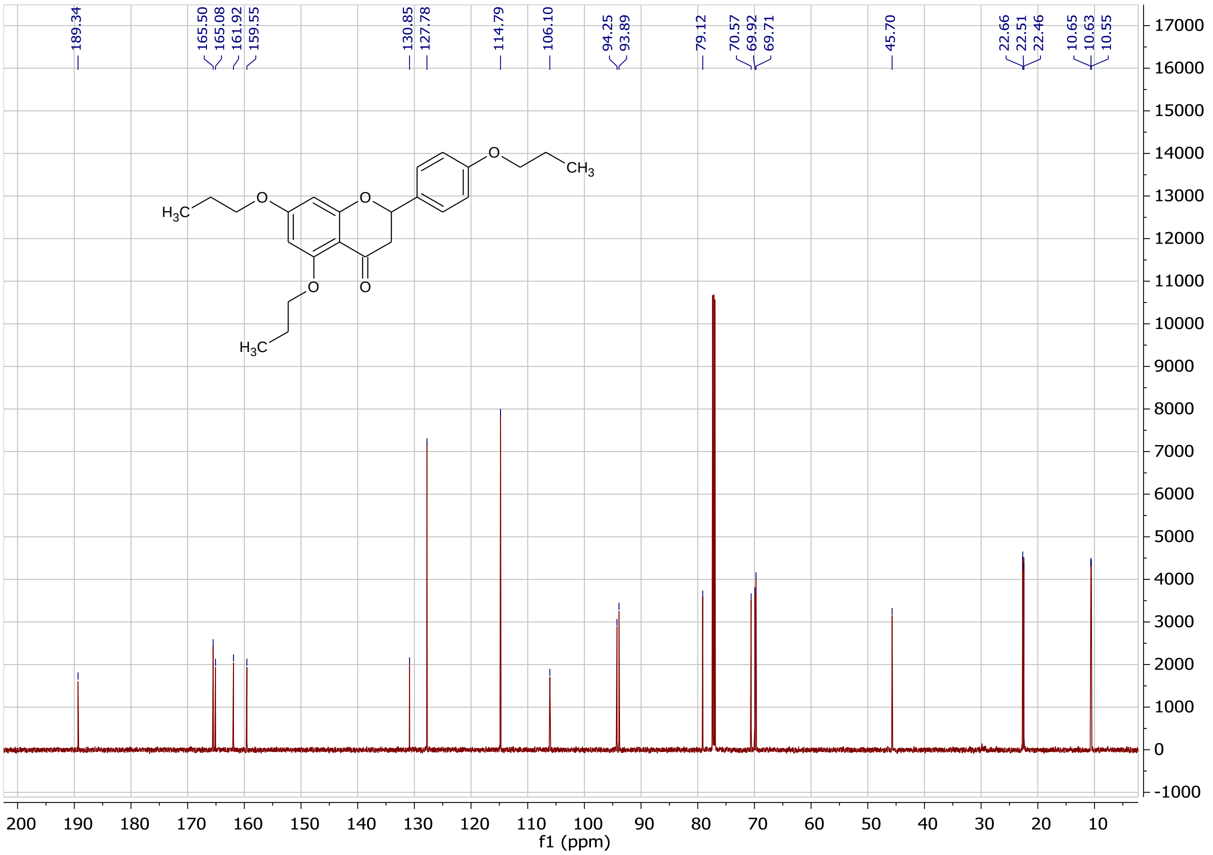

Fig S6. ^{13}C-NMR (150 MHz, $CDCl_3$) spectrum of 5,7,4′-Tri-*O*-propylnaringenin (**9a**)

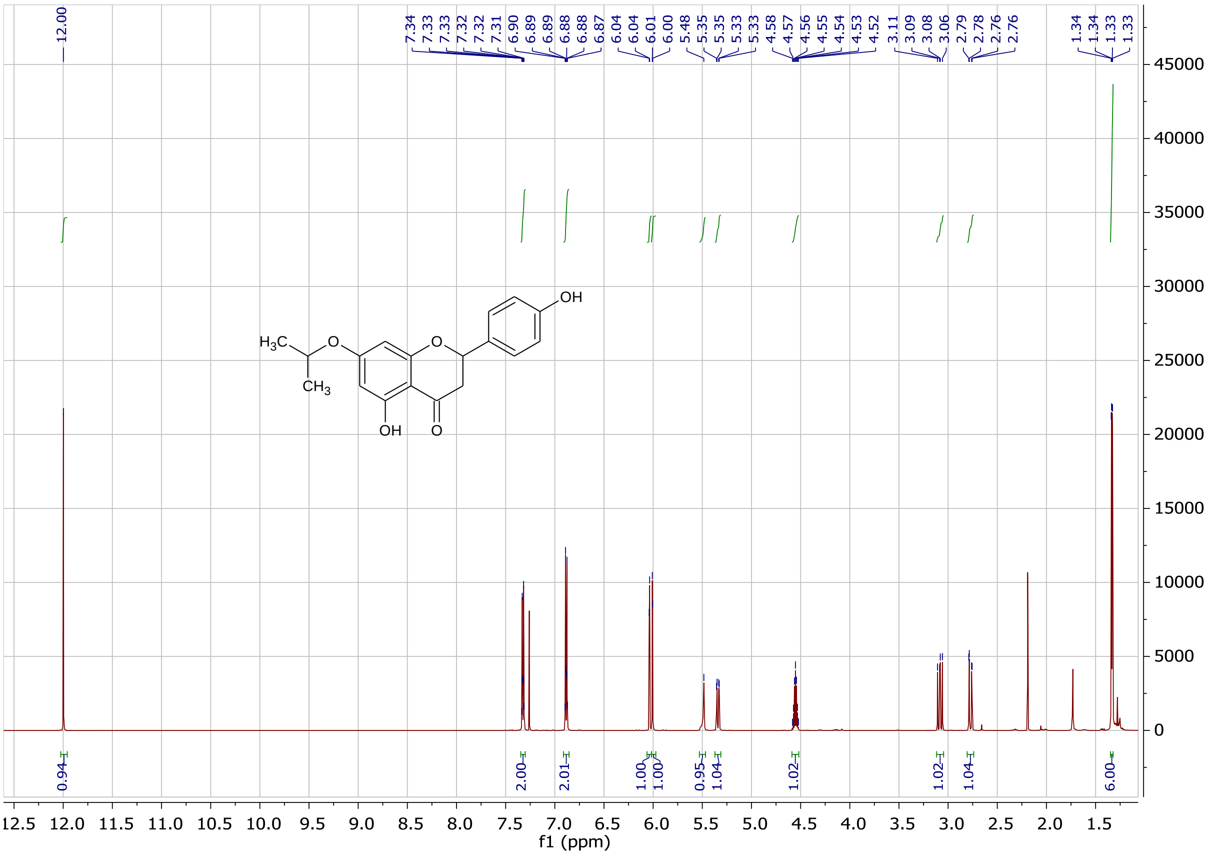

Fig S7. ^{1}H-NMR (600 MHz, $CDCl_3$) spectrum of 7-*O*-Isopropylnaringenin (**10a**)

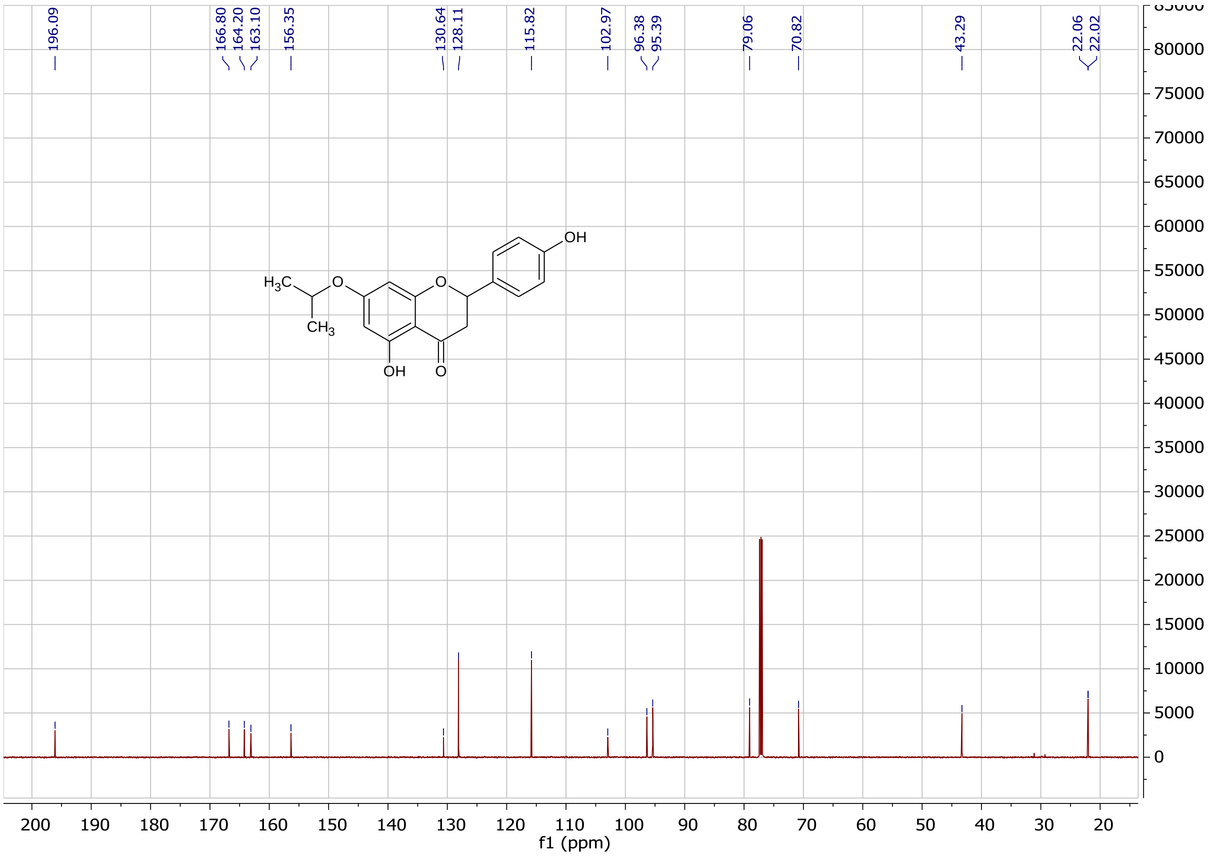

Fig S8. ^{13}C-NMR (150 MHz, $CDCl_3$) spectrum of 7-*O*-Isopropylnaringenin (**10a**)

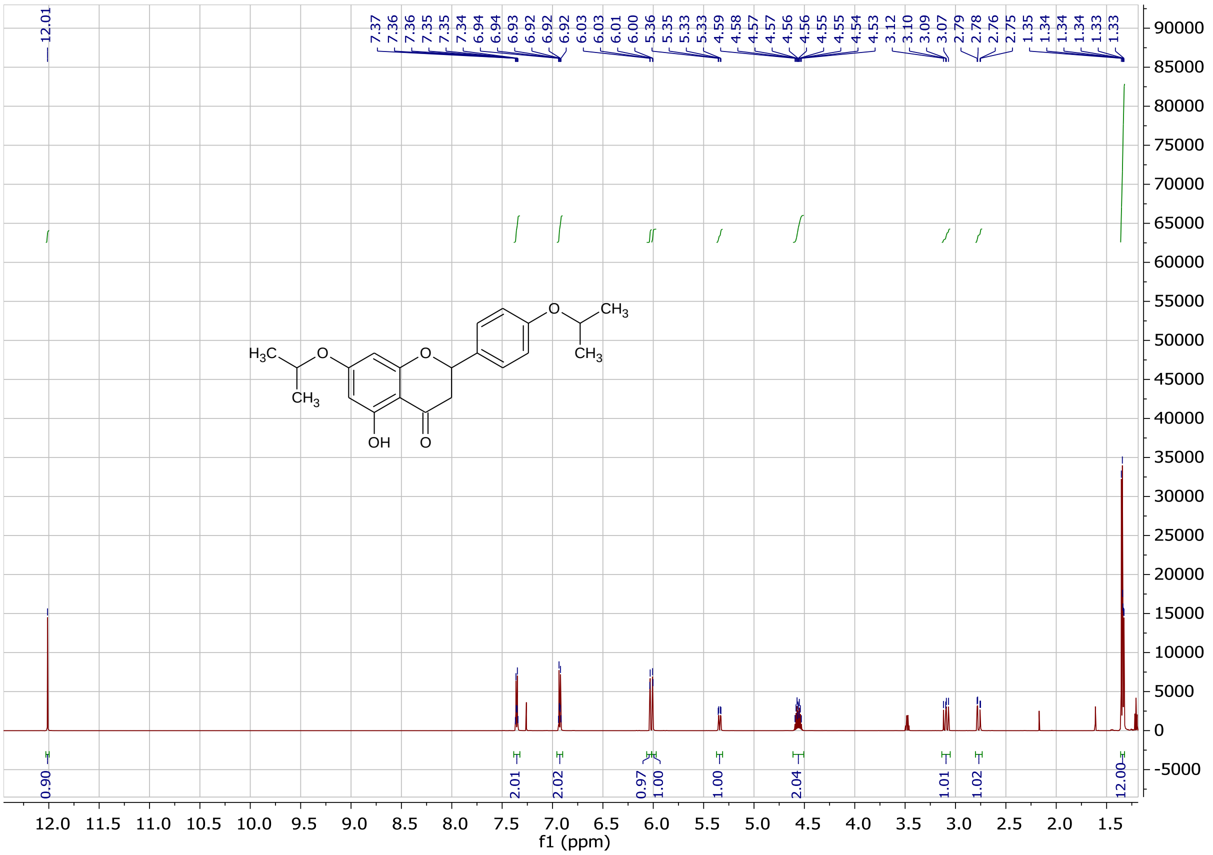

Fig S9. ^{1}H-NMR (600 MHz, $CDCl_3$) spectrum of 7,4′-Di-*O*-isopropylnaringenin (**11a**)

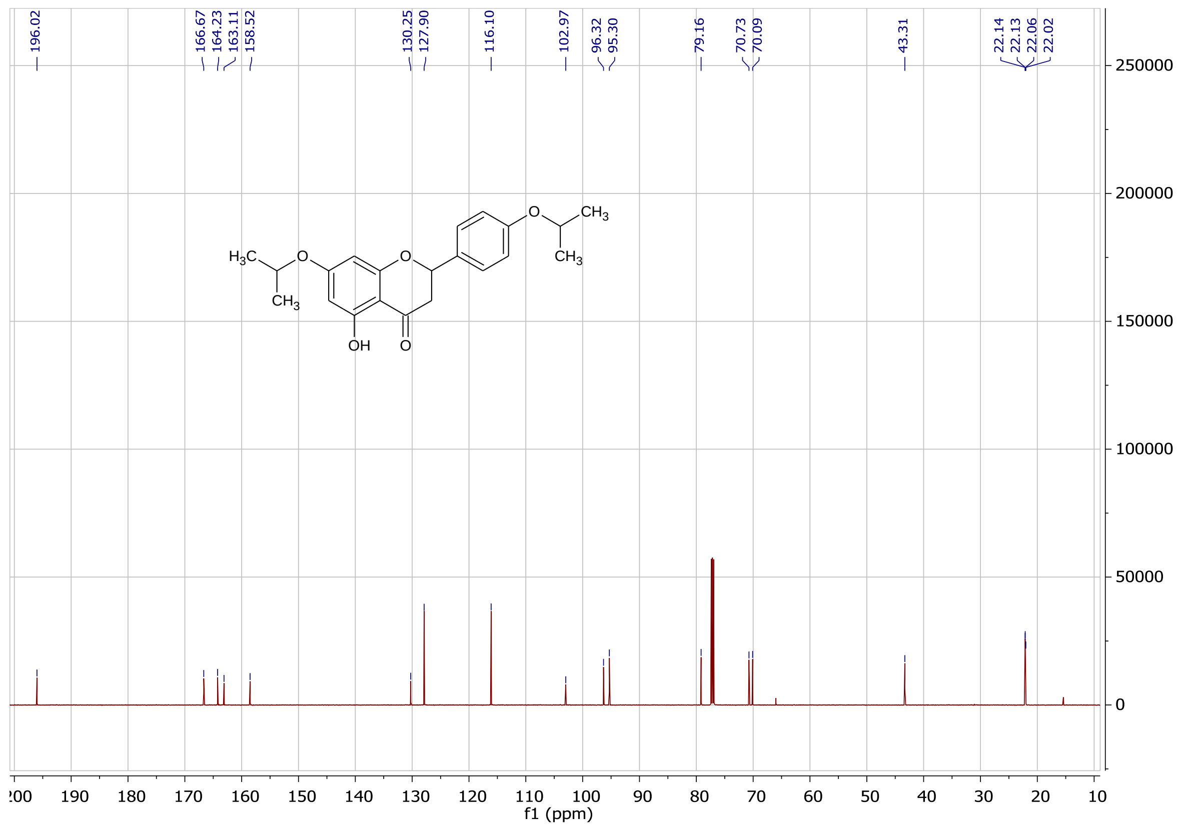

Fig S10. ^{13}C-NMR (150 MHz, $CDCl_3$) spectrum of 7,4′-Di-*O*-isopropylnaringenin (**11a**)

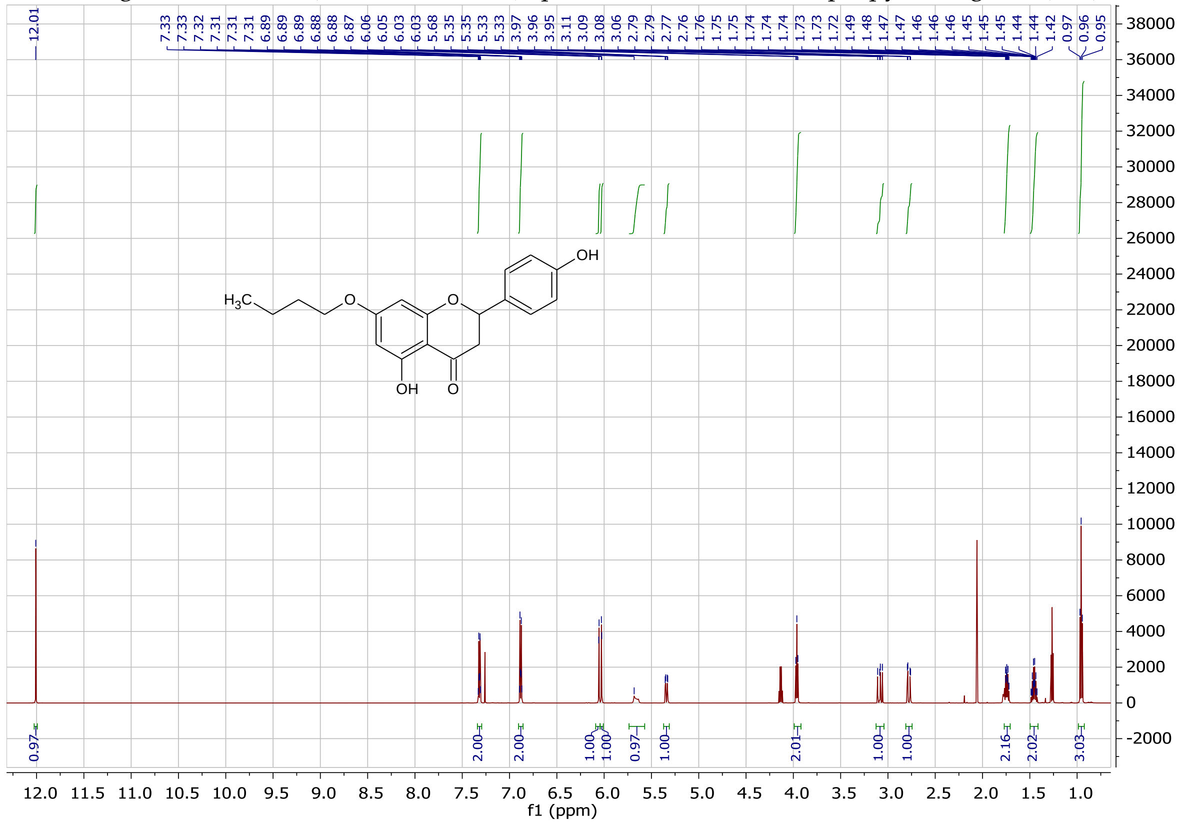

Fig S11. ^{1}H-NMR (600 MHz, $CDCl_3$) spectrum of 7-*O*-Butylnaringenin (**12a**)

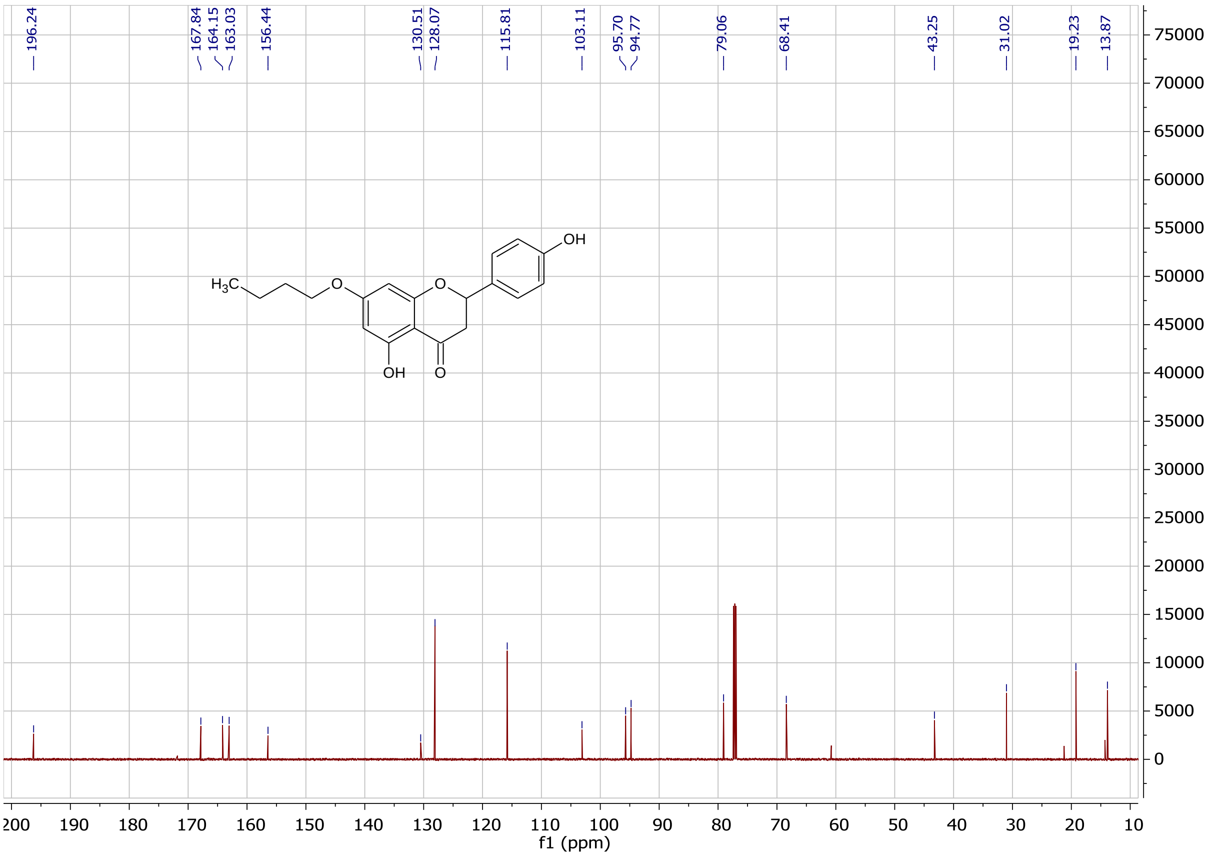

Fig S12. ^{13}C-NMR (150 MHz, $CDCl_3$) spectrum of 7-*O*-Butylnaringenin (**12a**)

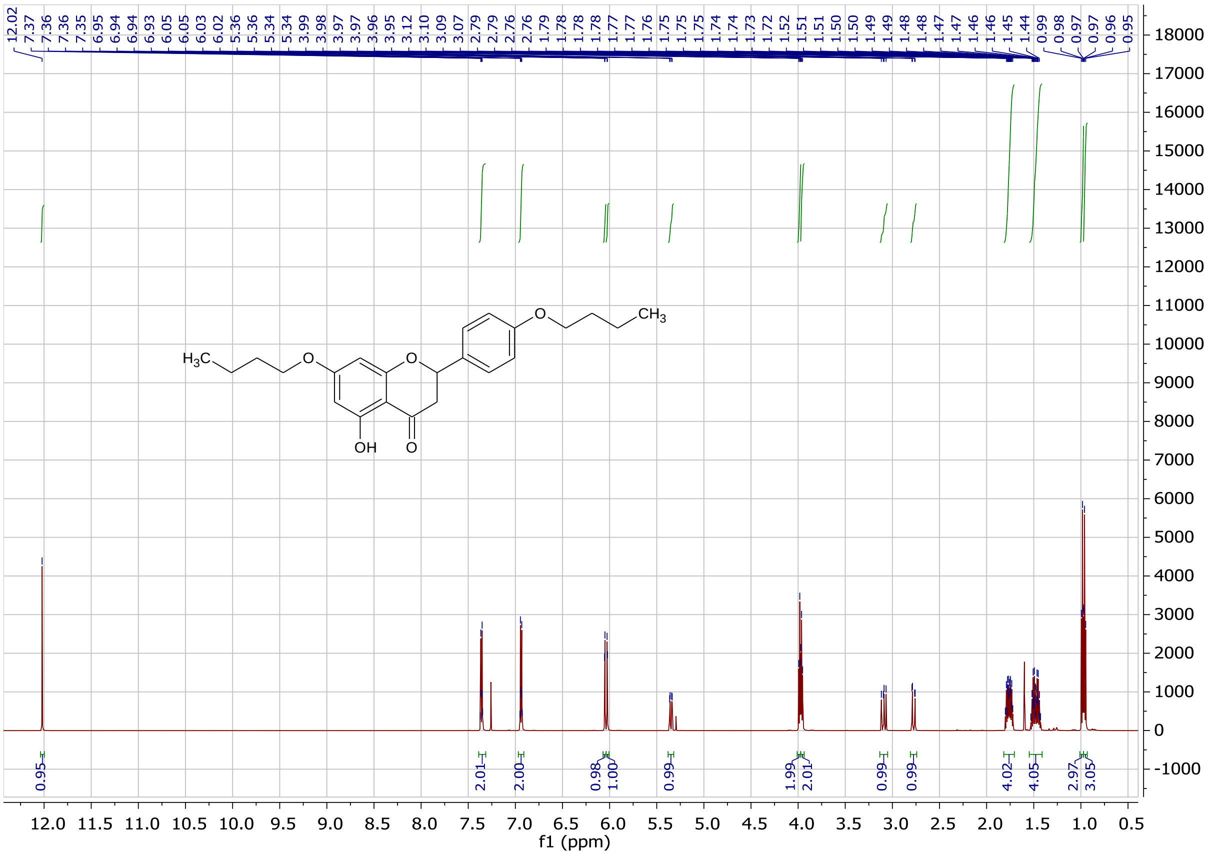

Fig S13. ^{1}H-NMR (600 MHz, $CDCl_3$) spectrum of 7,4′-Di-*O*-butylnaringenin (**13a**)

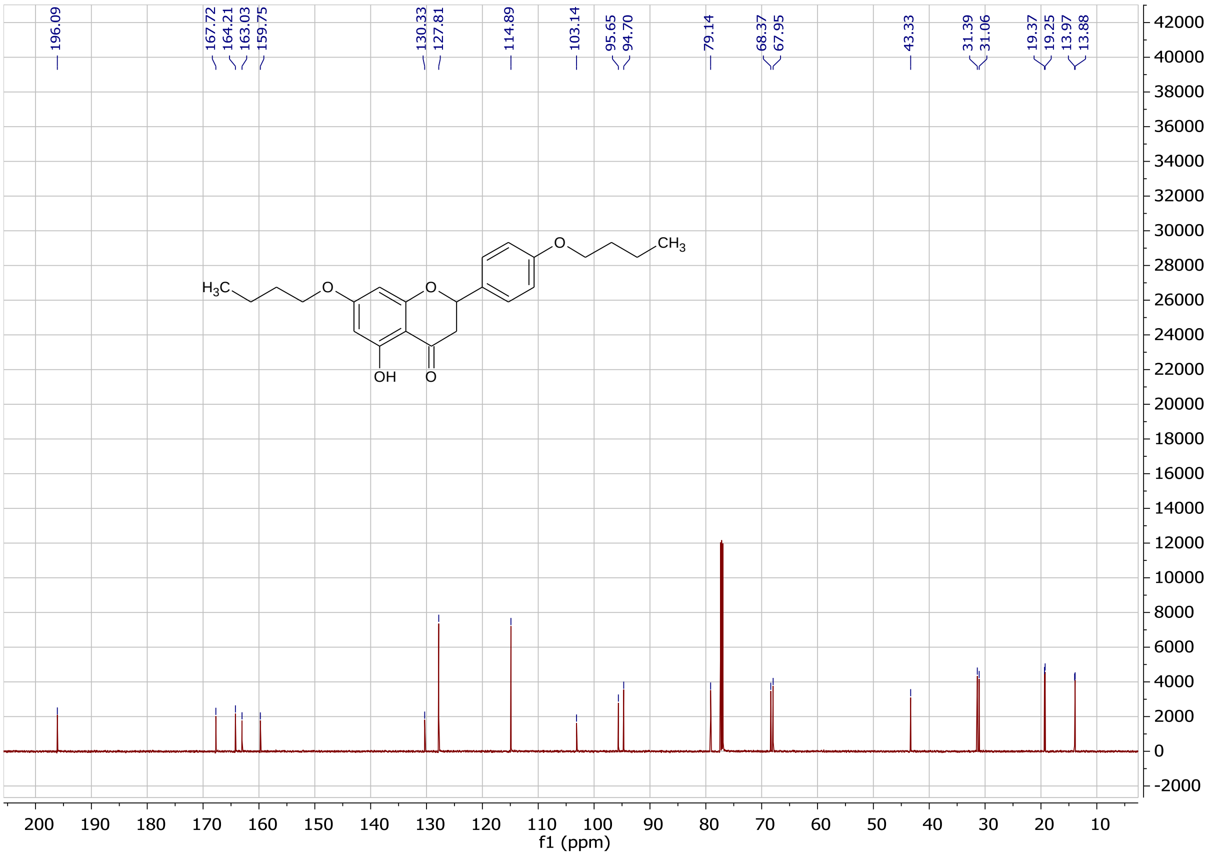

Fig S14. ^{13}C-NMR (150 MHz, $CDCl_3$) spectrum of 7,4′-Di-*O*-butylnaringenin (**13a**)

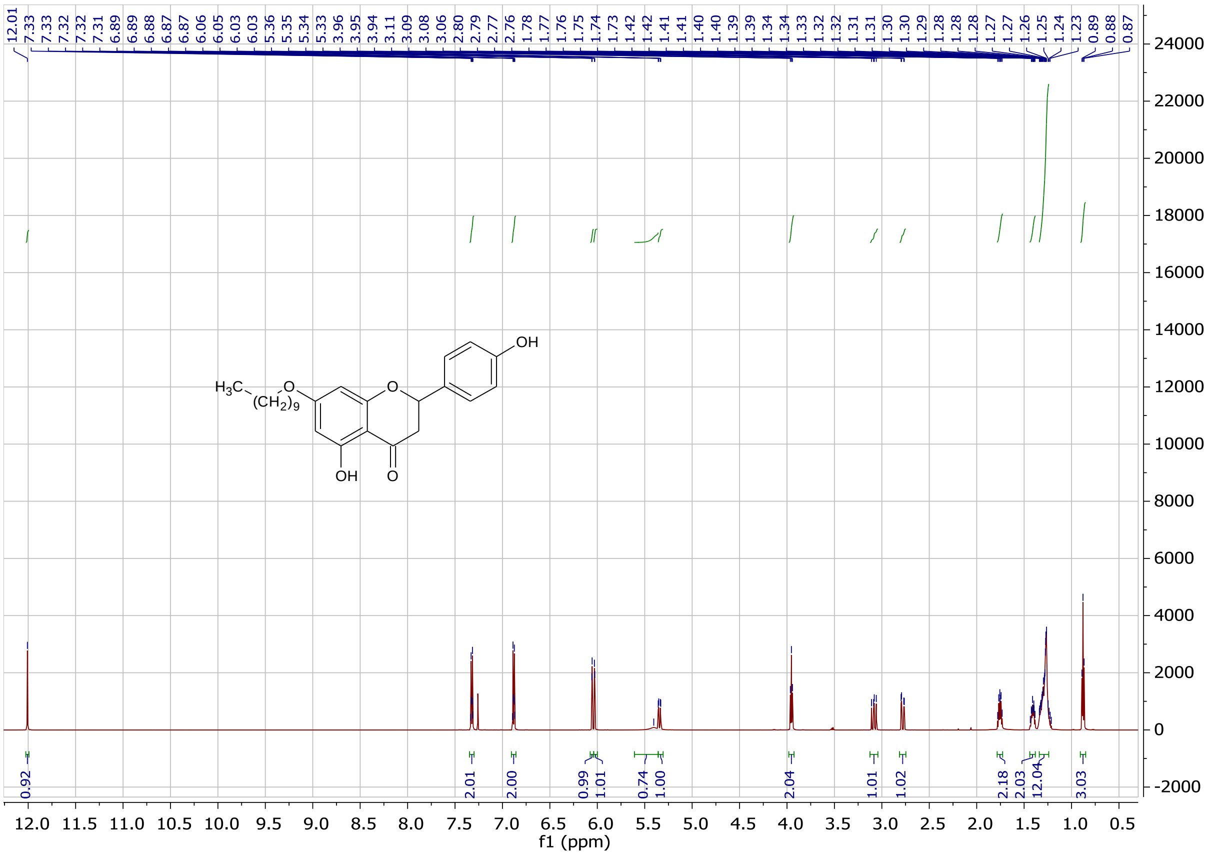

Fig S15. ^{1}H-NMR (600 MHz, $CDCl_3$) spectrum of 7-*O*-Decylnaringenin (**16a**)

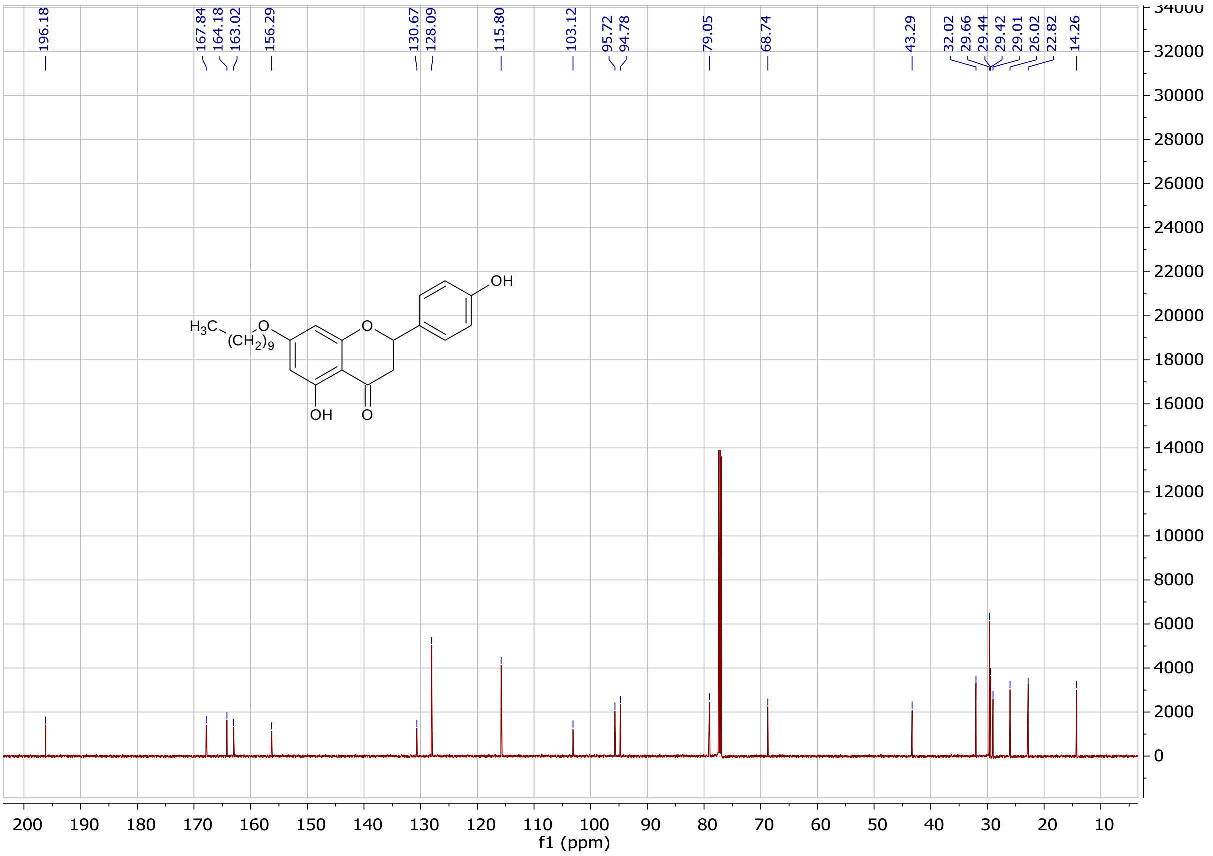

Fig S16. ^{13}C-NMR (150 MHz, $CDCl_3$) spectrum of 7-*O*-Decylnaringenin (**16a**)

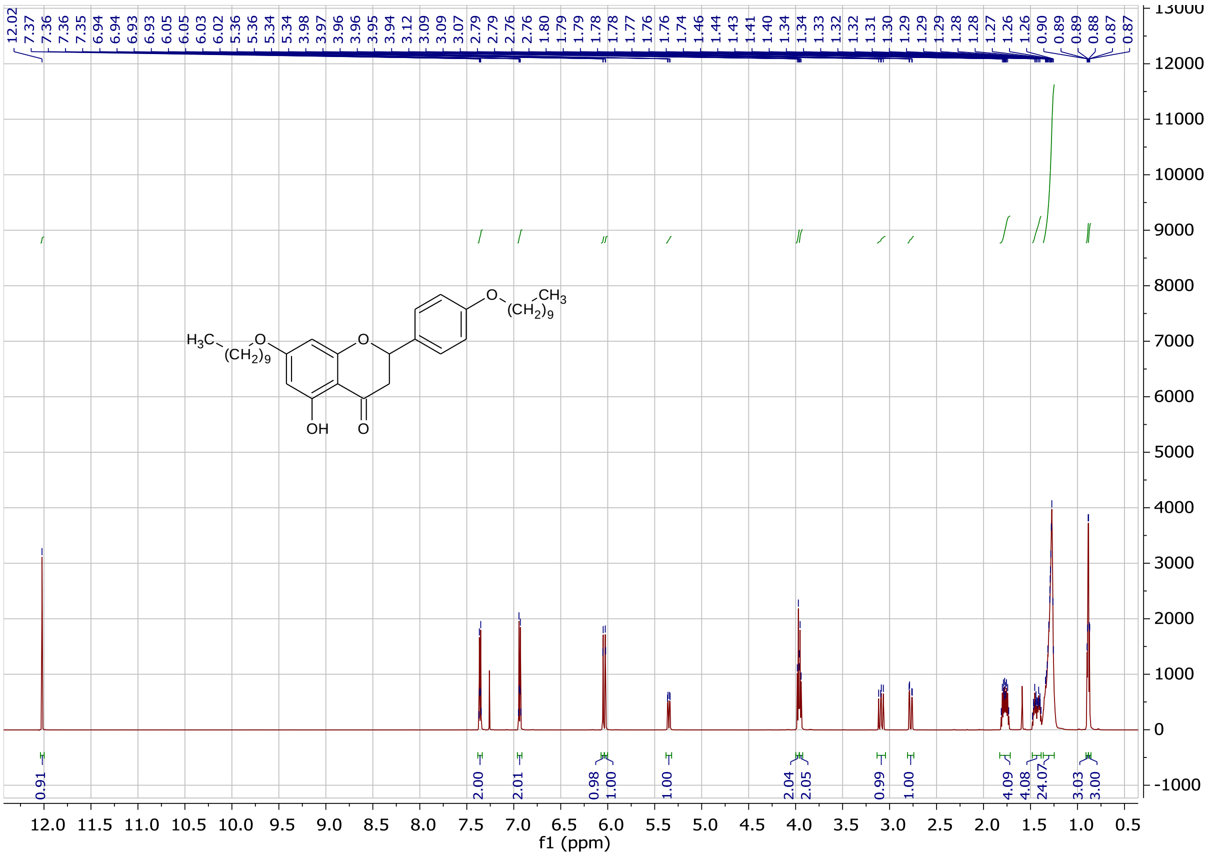

Fig S17. ^{1}H-NMR (600 MHz, $CDCl_3$) spectrum of 7,4′-Di-*O*-decylnaringenin (**17a**)

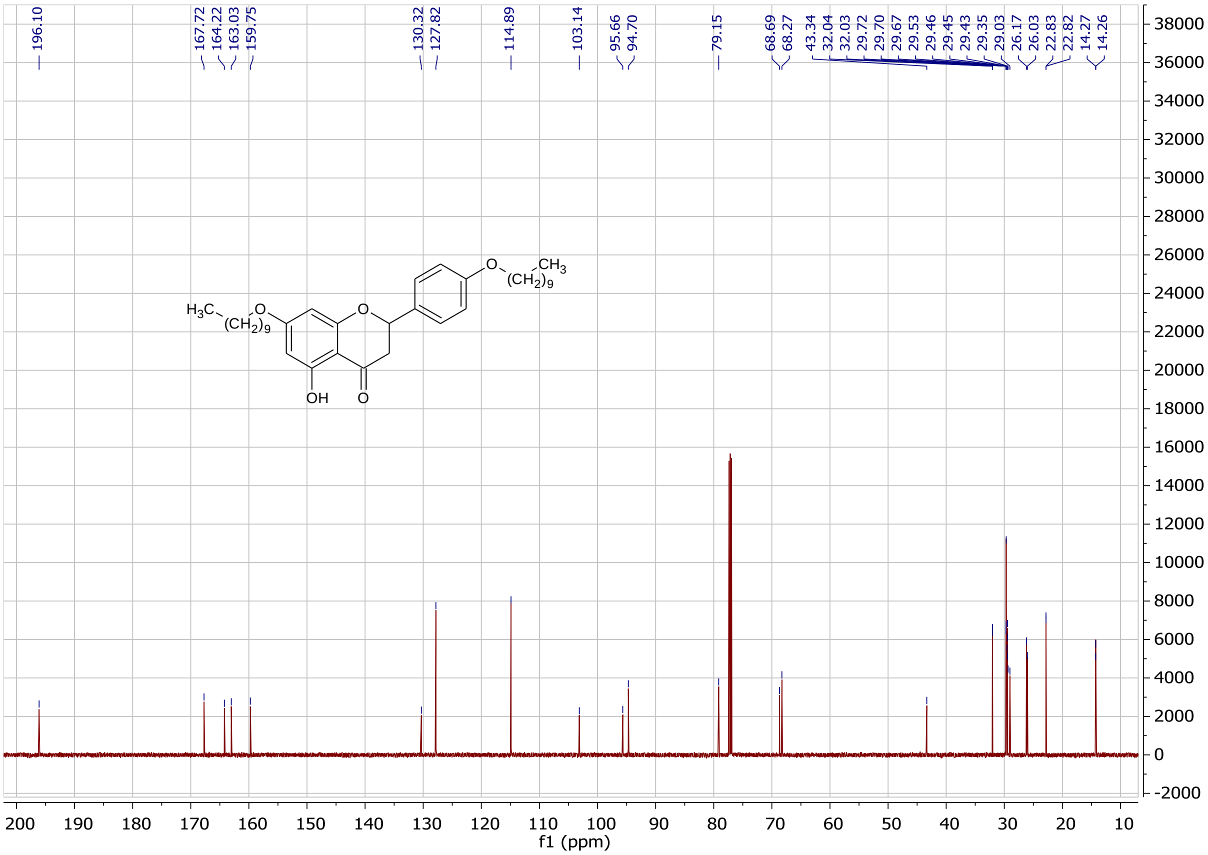

Fig S18. ^{13}C-NMR (150 MHz, $CDCl_3$) spectrum of 7,4′-Di-*O*-decylnaringenin (**17a**)

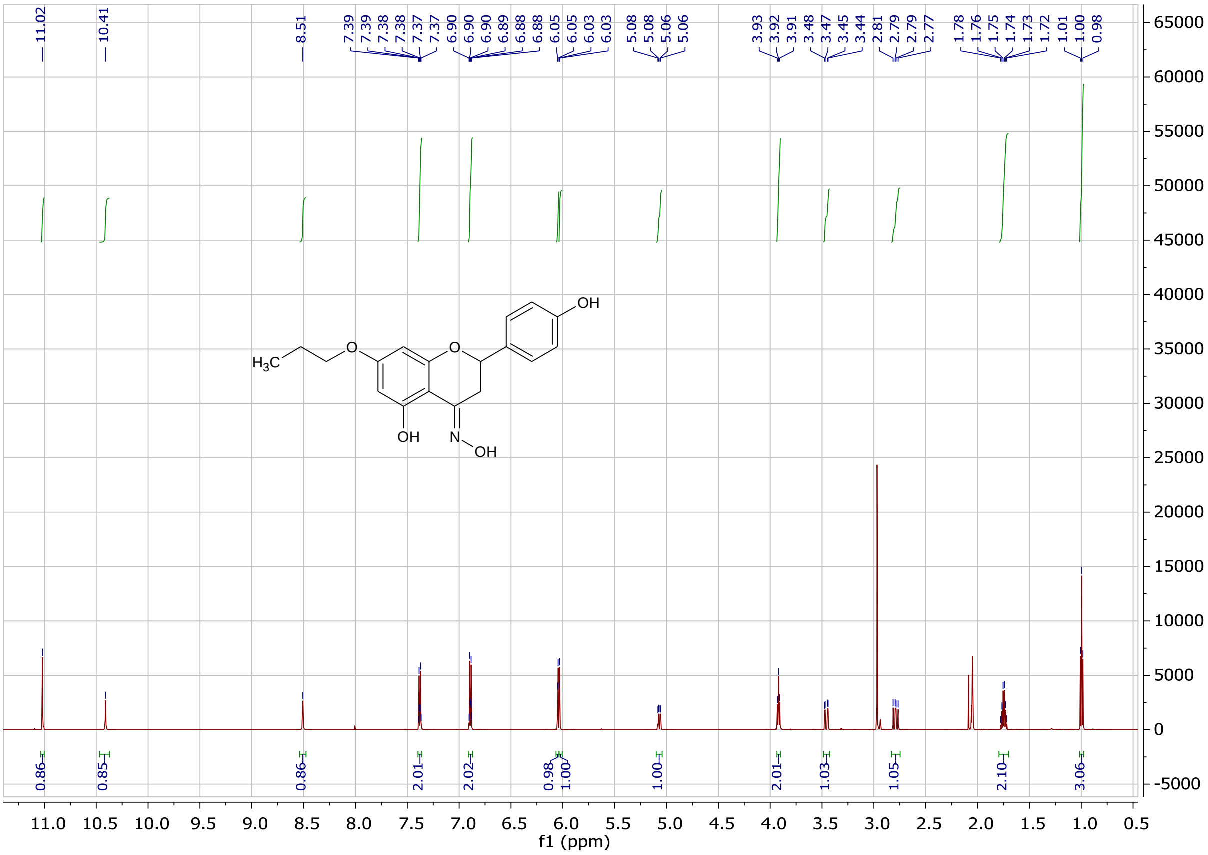

Fig S19. ^{1}H-NMR (600 MHz, $(CD_3)_2CO$) spectrum of 7-*O*-Propylnaringenin oxime (**7b**)

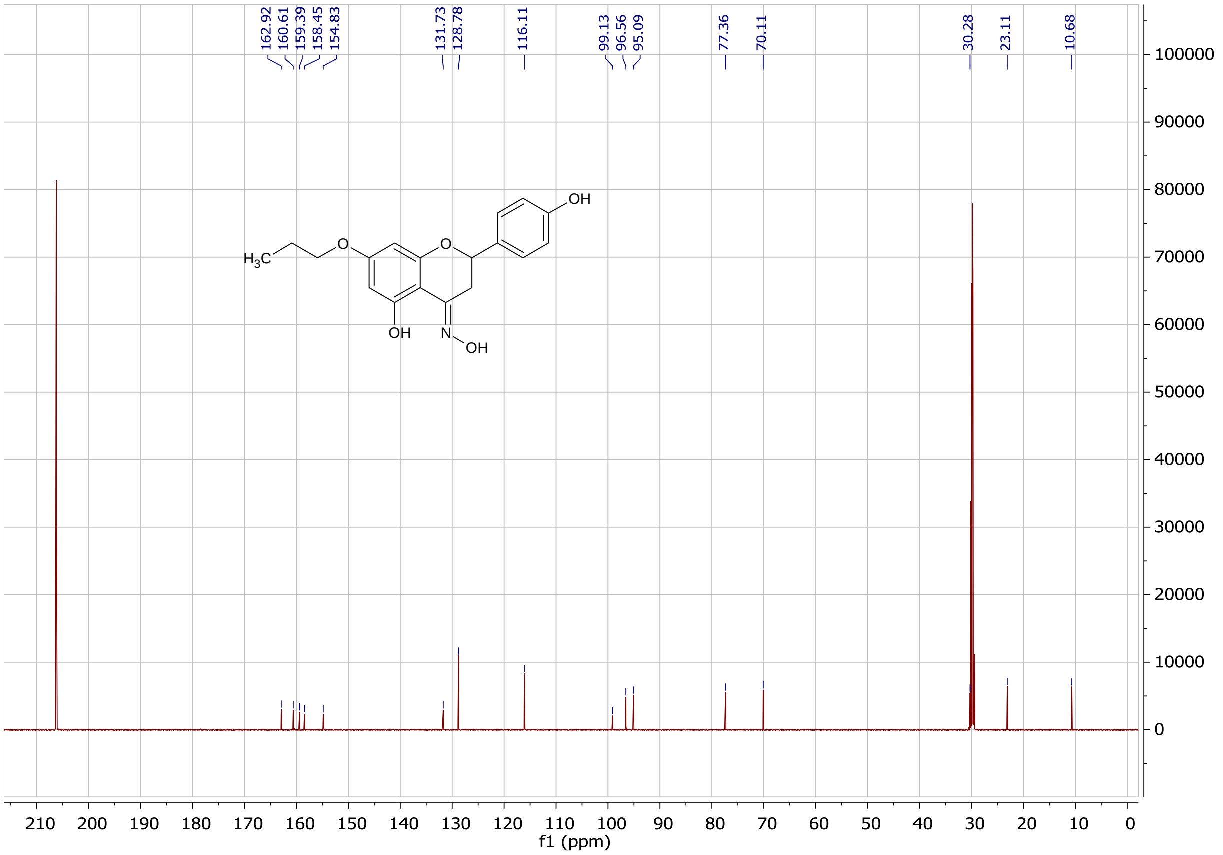

Fig S20. ^{13}C-NMR (150 MHz, $(CD_3)_2CO$) spectrum of 7-*O*-Propylnaringenin oxime (**7b**)

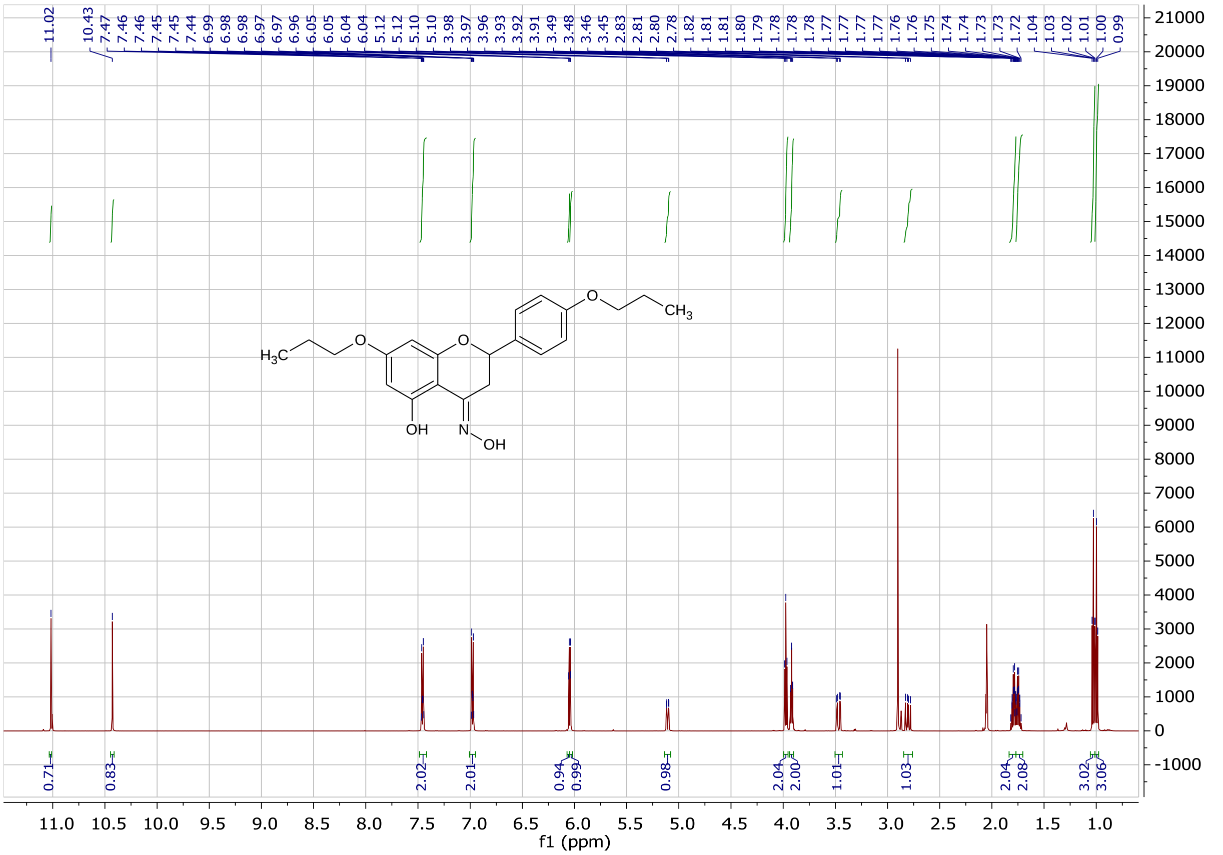

Fig S21. ^{1}H-NMR (600 MHz, $(CD_3)_2CO$) spectrum of 7,4′-Di-*O*-propylnaringenin oxime (**8b**)

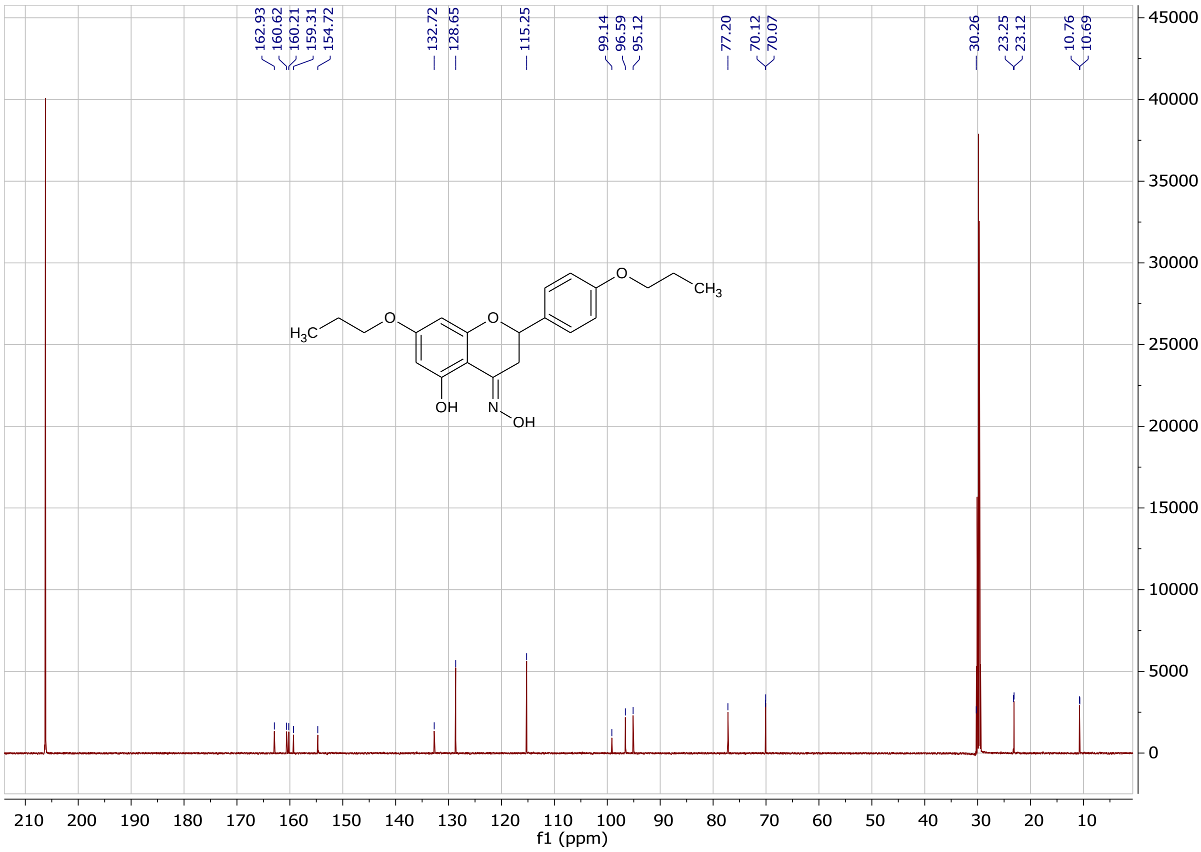

Fig S22. ^{13}C-NMR (150 MHz, $(CD_3)_2CO$) spectrum of 7,4′-Di-*O*-propylnaringenin oxime (**8b**)

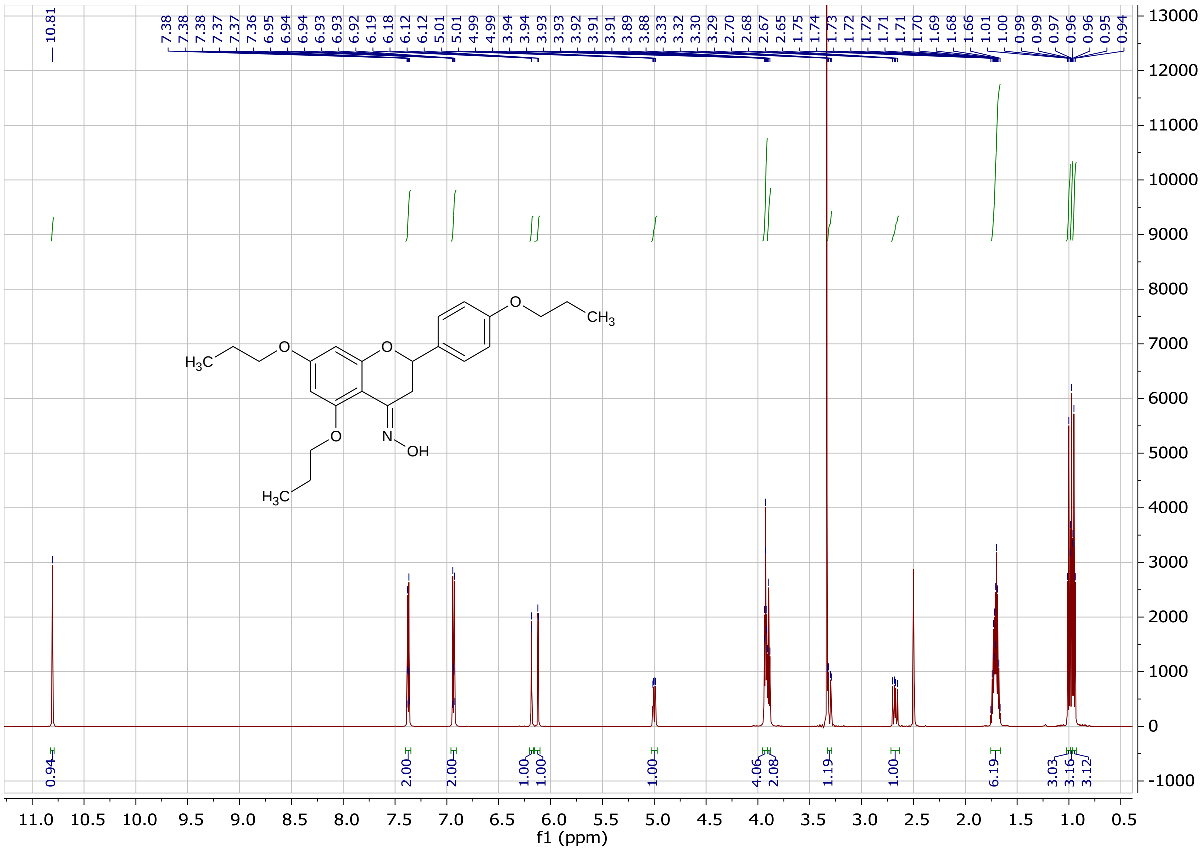

Fig S23. ^{1}H-NMR (600 MHz, $(CD_3)_2SO$) spectrum of 5,7,4′-Tri-*O*-propylnaringenin oxime (**9b**)

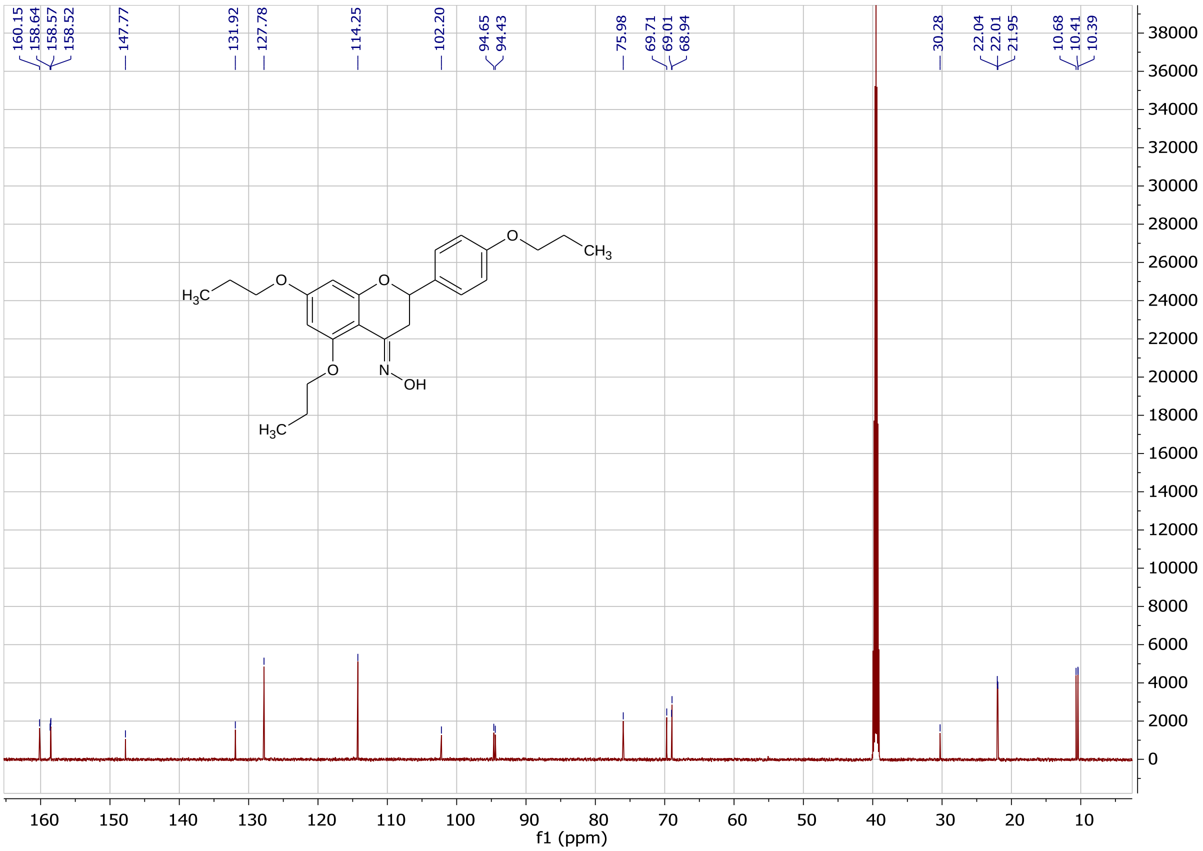

Fig S24. ^{13}C-NMR (150 MHz, $(CD_3)_2SO$) spectrum of 5,7,4′-Tri-*O*-propylnaringenin oxime (**9b**)

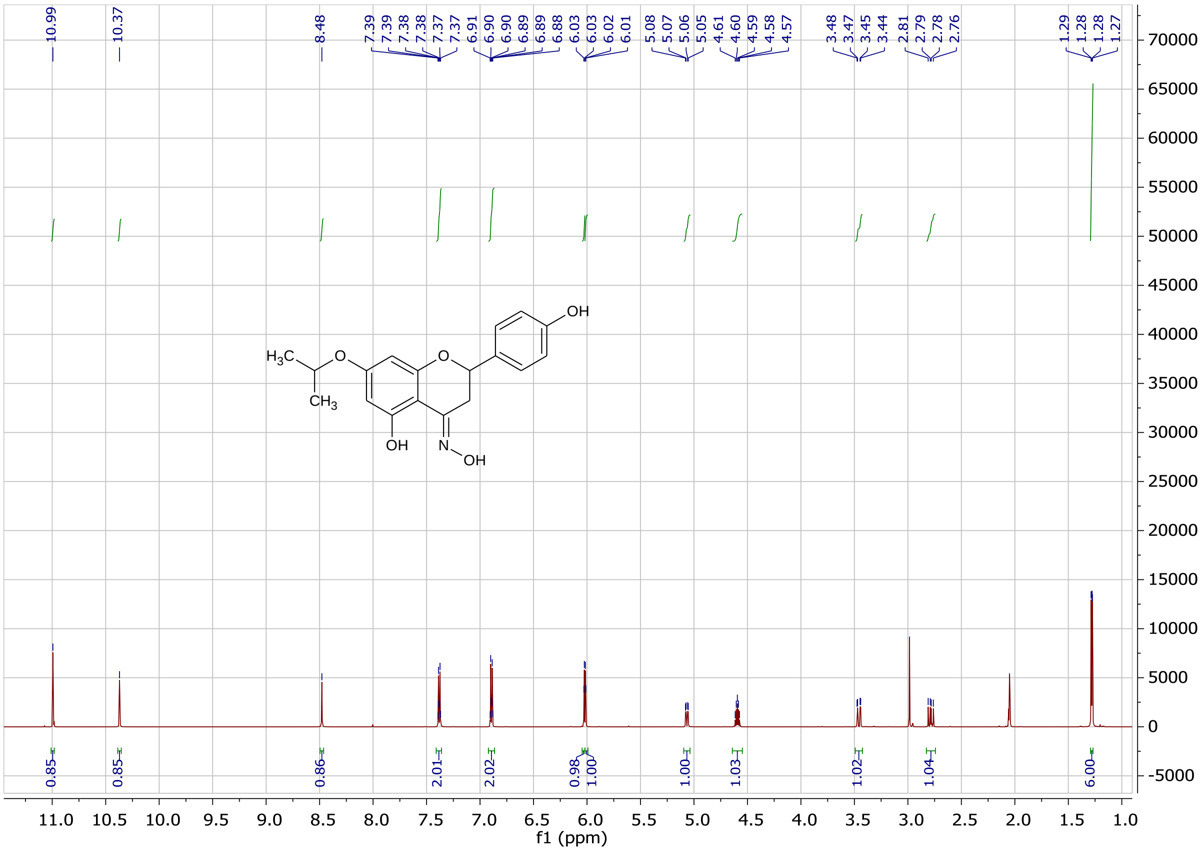

Fig S25. ^{1}H-NMR (600 MHz, $(CD_3)_2CO$) spectrum of 7-*O*-Isopropylnaringenin oxime (**10b**)

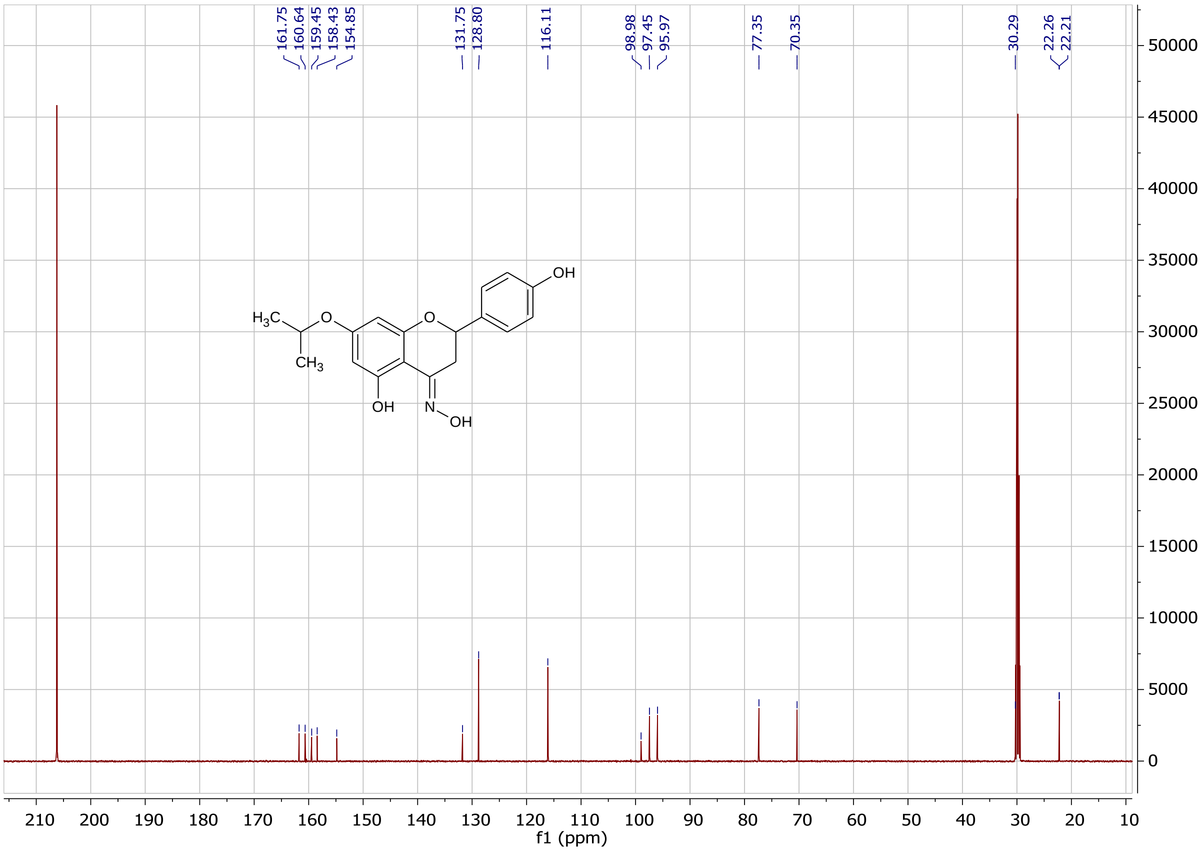

Fig S26. ^{13}C-NMR (150 MHz, $(CD_3)_2CO$) spectrum of 7-*O*-Isopropylnaringenin oxime (**10b**)

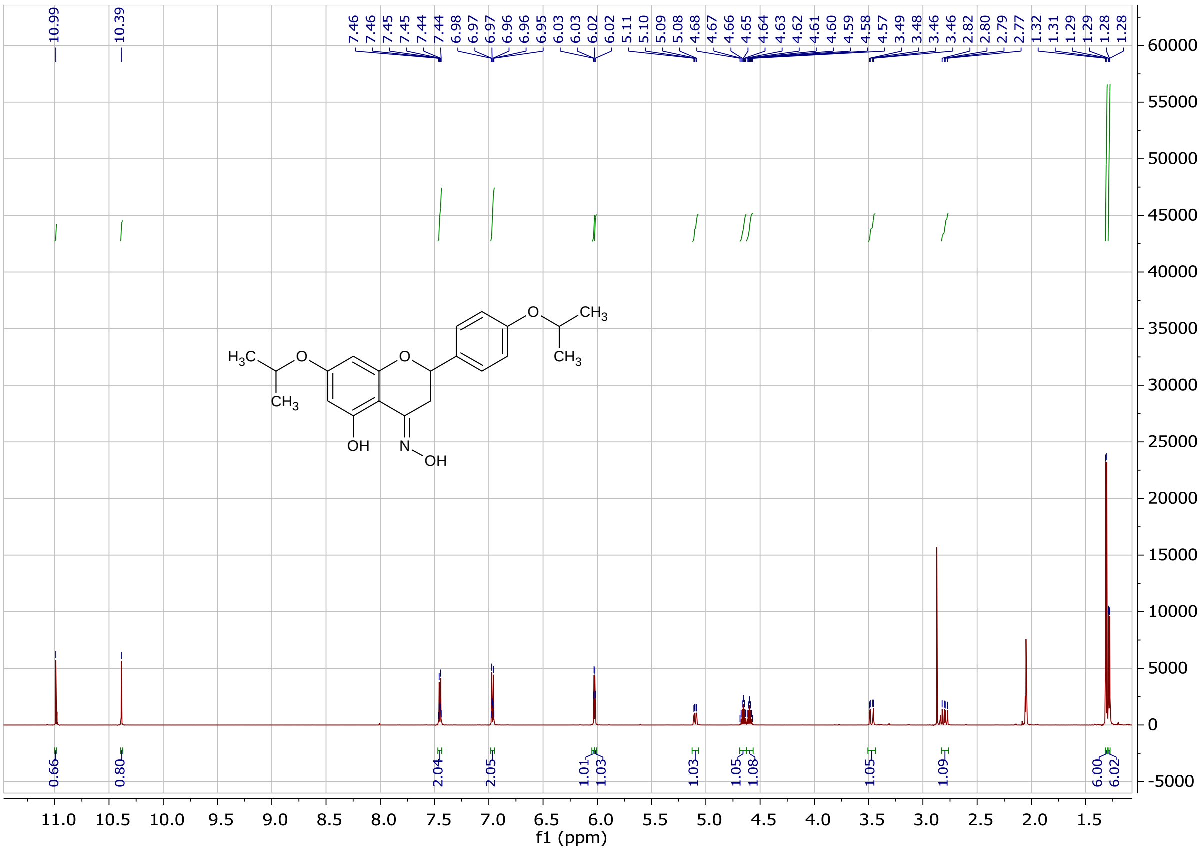

Fig S27. ^{1}H-NMR (600 MHz, $(CD_3)_2CO$) spectrum of 7,4′-Di-*O*-isopropylnaringenin oxime (**11b**)

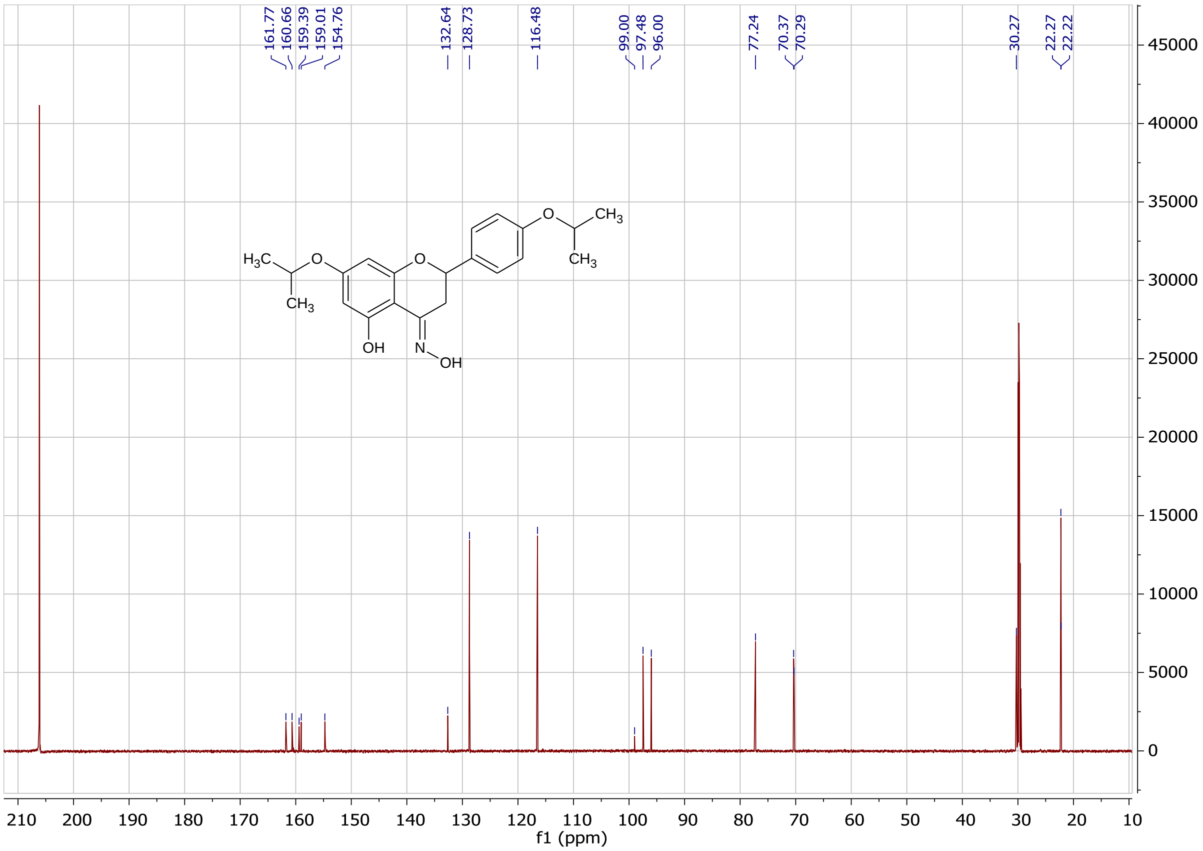

Fig S28. ^{13}C-NMR (150 MHz, $(CD_3)_2CO$) spectrum of 7,4′-Di-*O*-isopropylnaringenin oxime (**11b**)

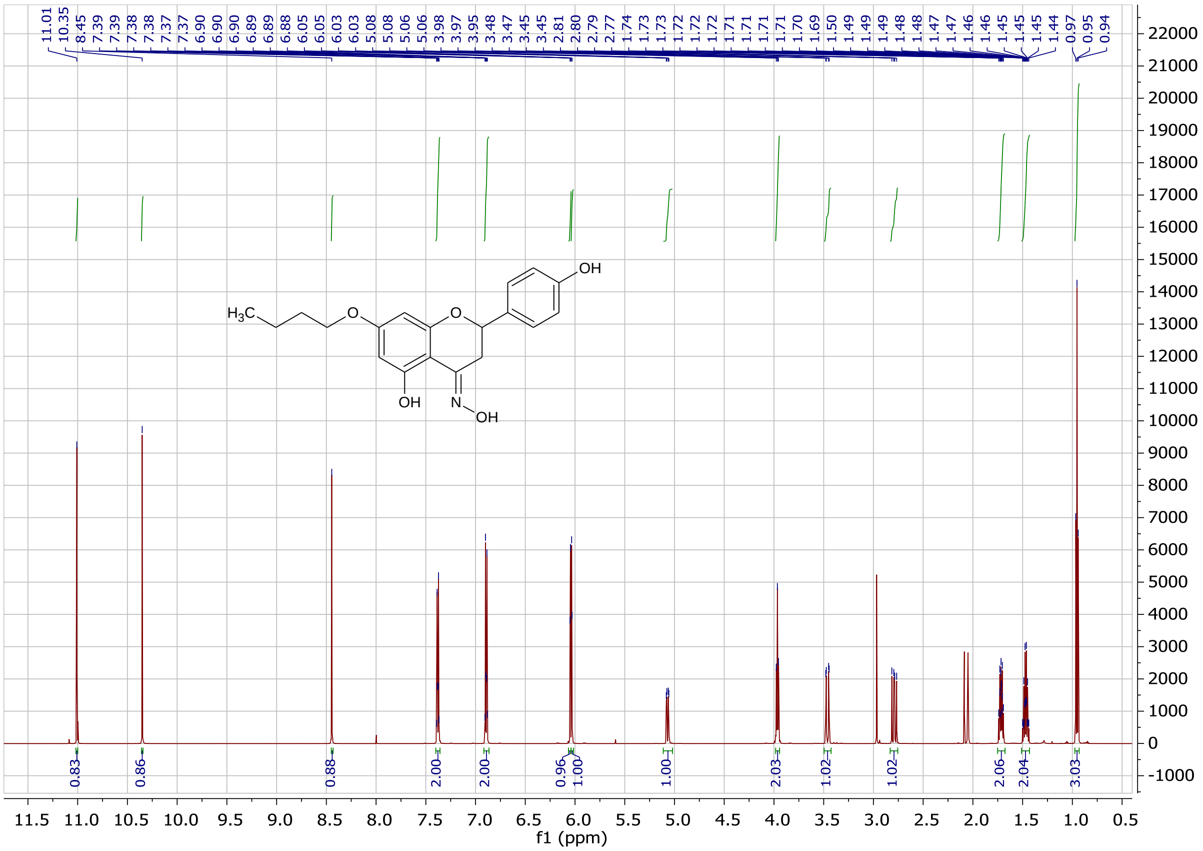

Fig S29. ^{1}H-NMR (600 MHz, $(CD_3)_2CO$) spectrum of 7-*O*-Butylnaringenin oxime (**12b**)

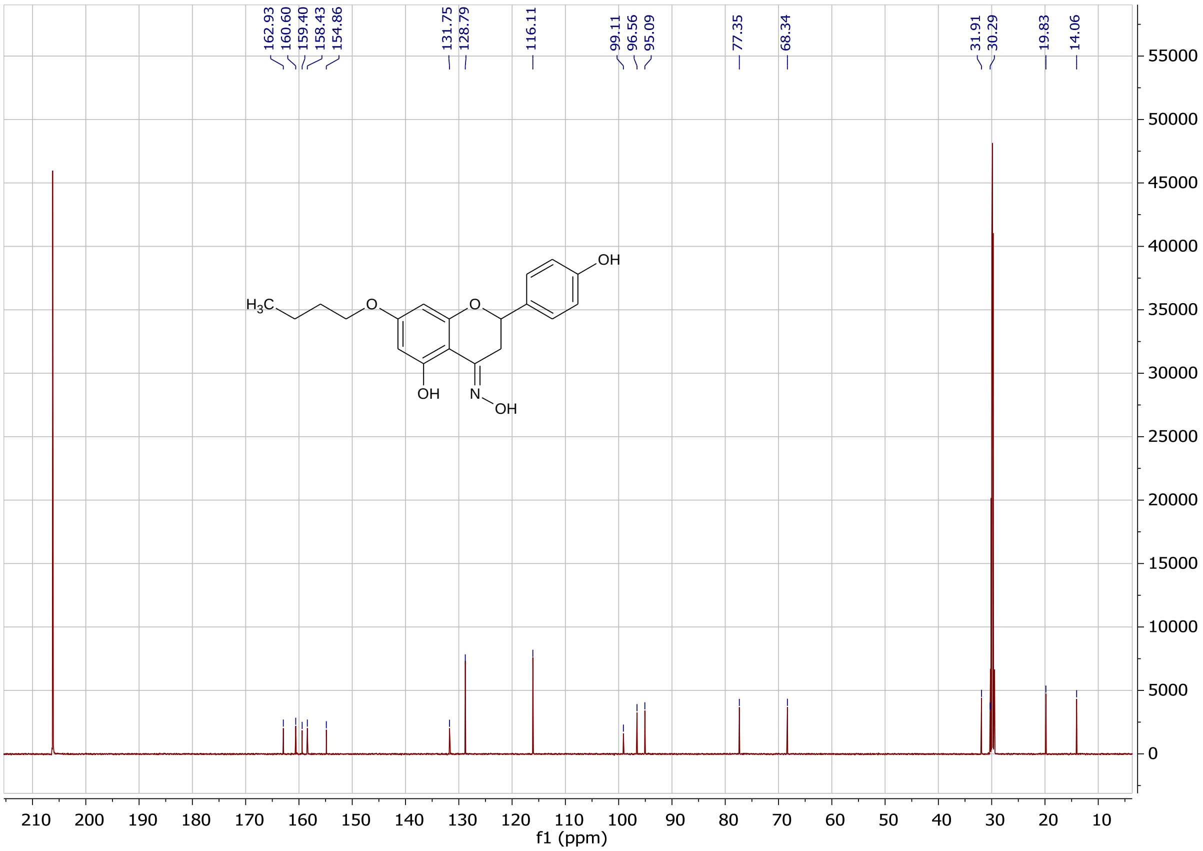

Fig S30. ^{13}C-NMR (150 MHz, $(CD_3)_2CO$) spectrum of 7-*O*-Butylnaringenin oxime (**12b**)

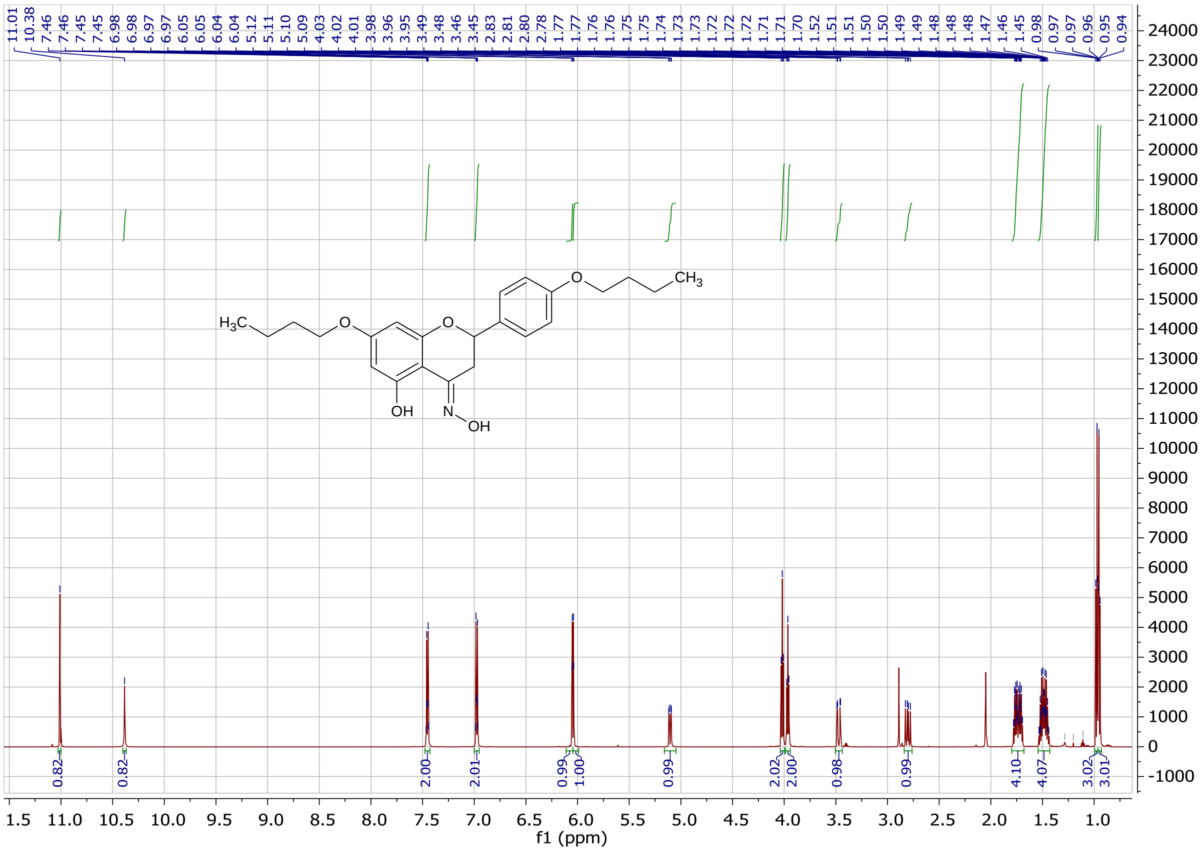

Fig S31. ^{1}H-NMR (600 MHz, $(CD_3)_2CO$) spectrum of 7,4′-Di-*O*-butylnaringenin oxime (**13b**)

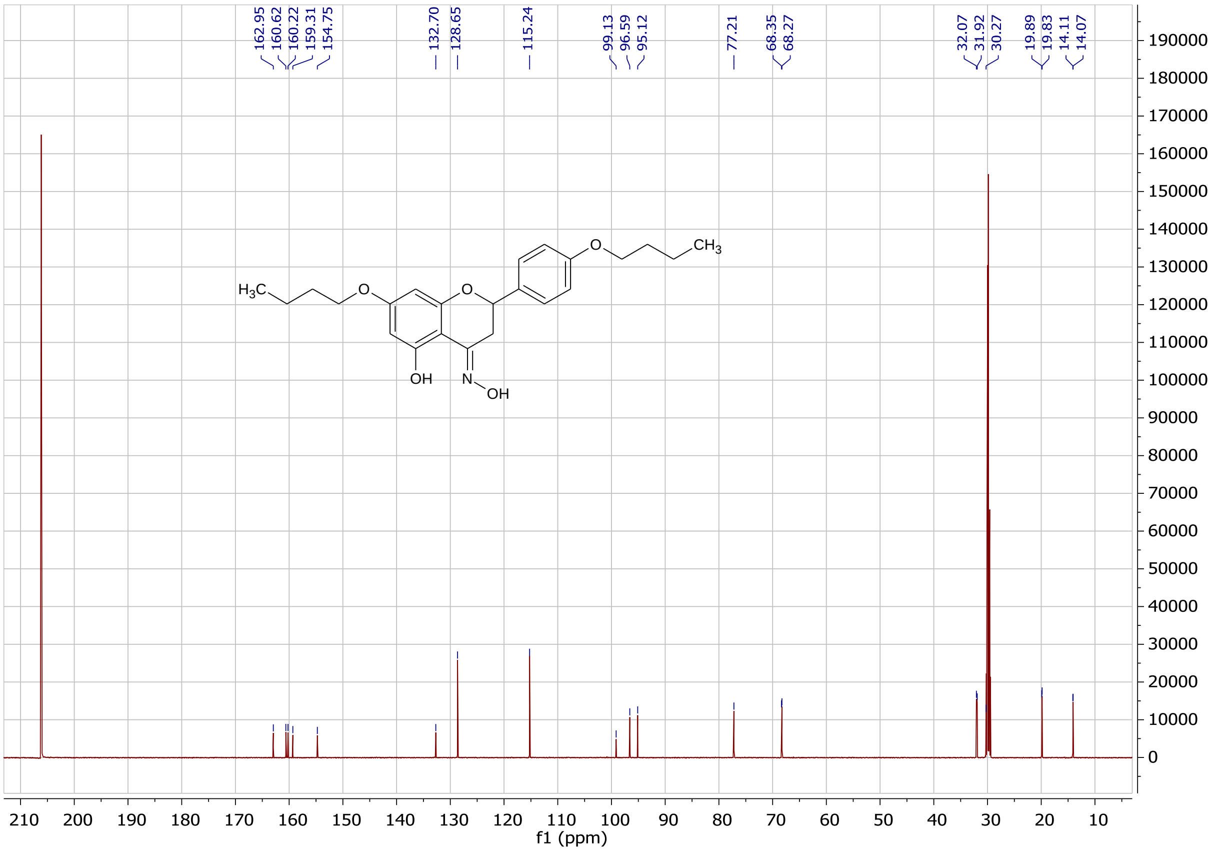

Fig S32. ^{13}C-NMR (150 MHz, $(CD_3)_2CO$) spectrum of 7,4′-Di-*O*-butylnaringenin oxime (**13b**)

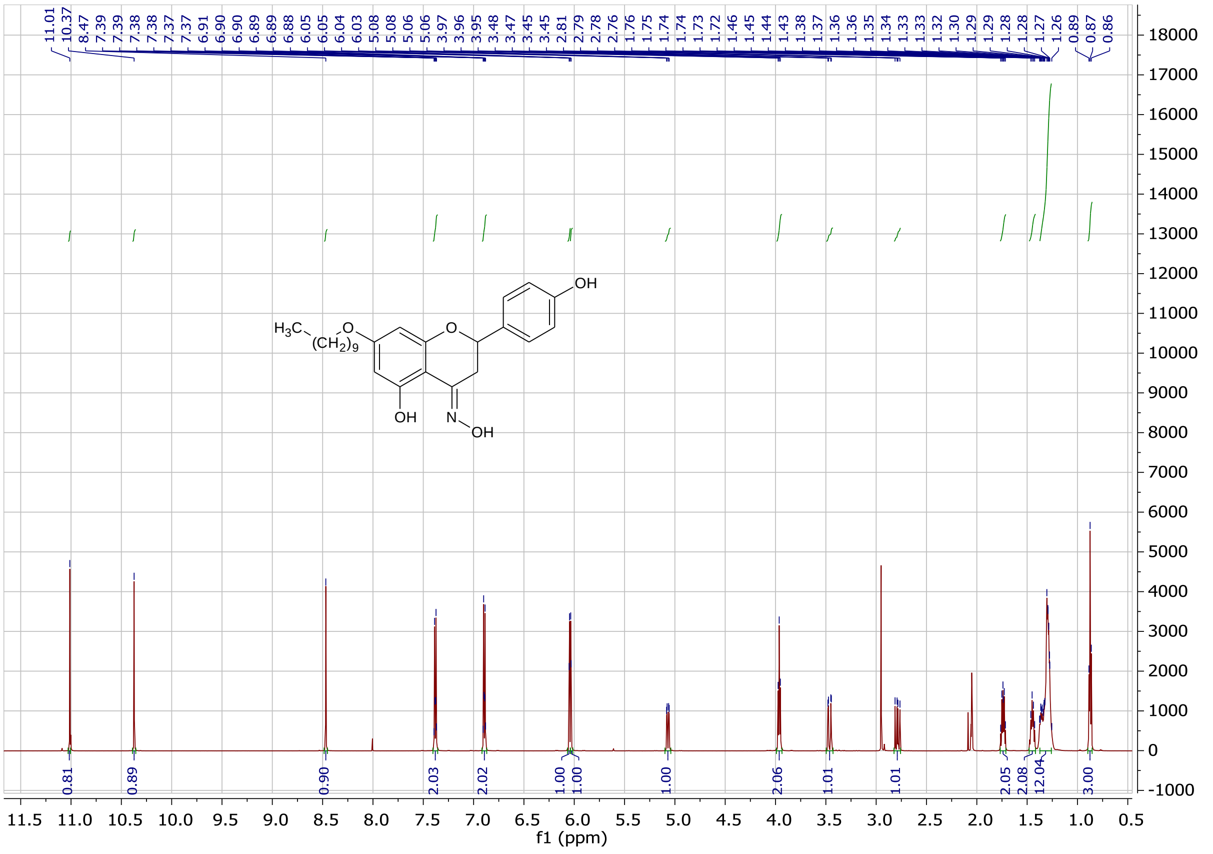

Fig S33. ^{1}H-NMR (600 MHz, $(CD_3)_2CO$) spectrum of 7-*O*-Decylnaringenin oxime (**16b**)

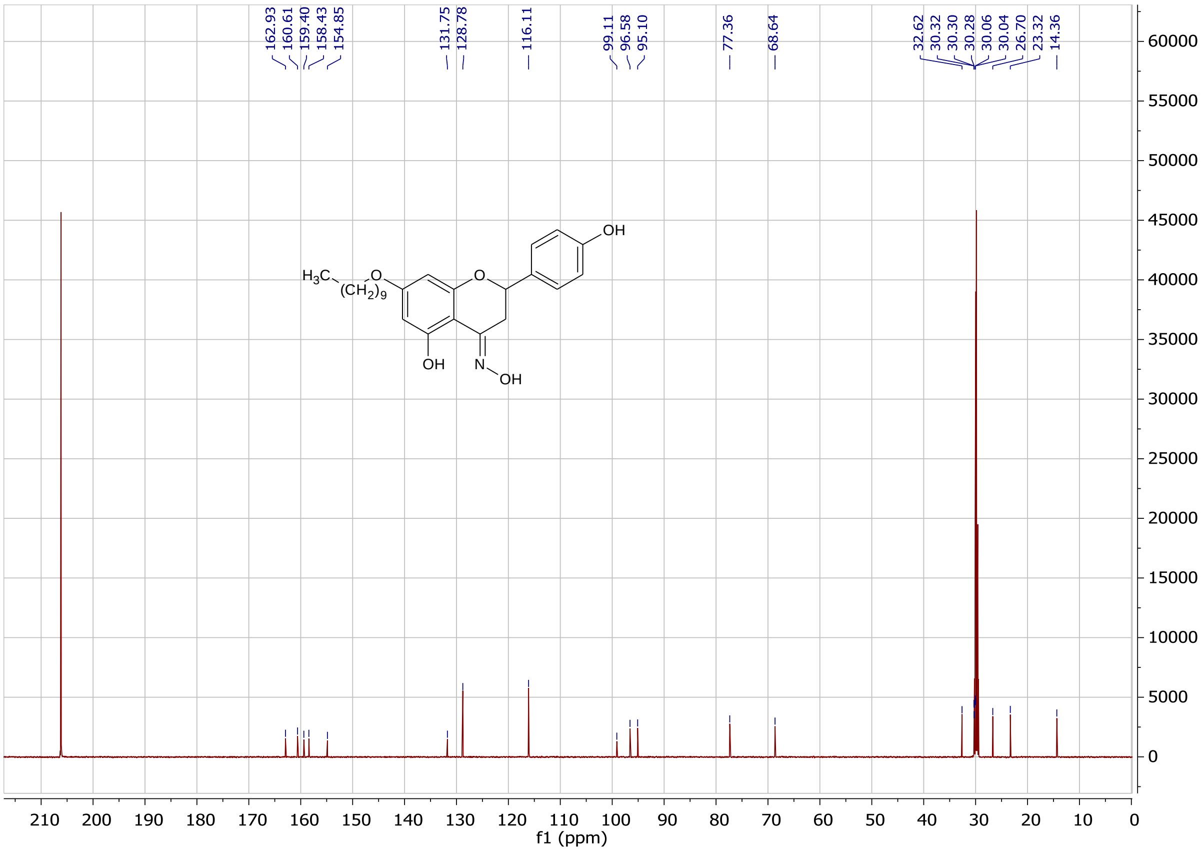

Fig S34. ^{13}C-NMR (150 MHz, $(CD_3)_2CO$) spectrum of 7-*O*-Decylnaringenin oxime (**16b**)

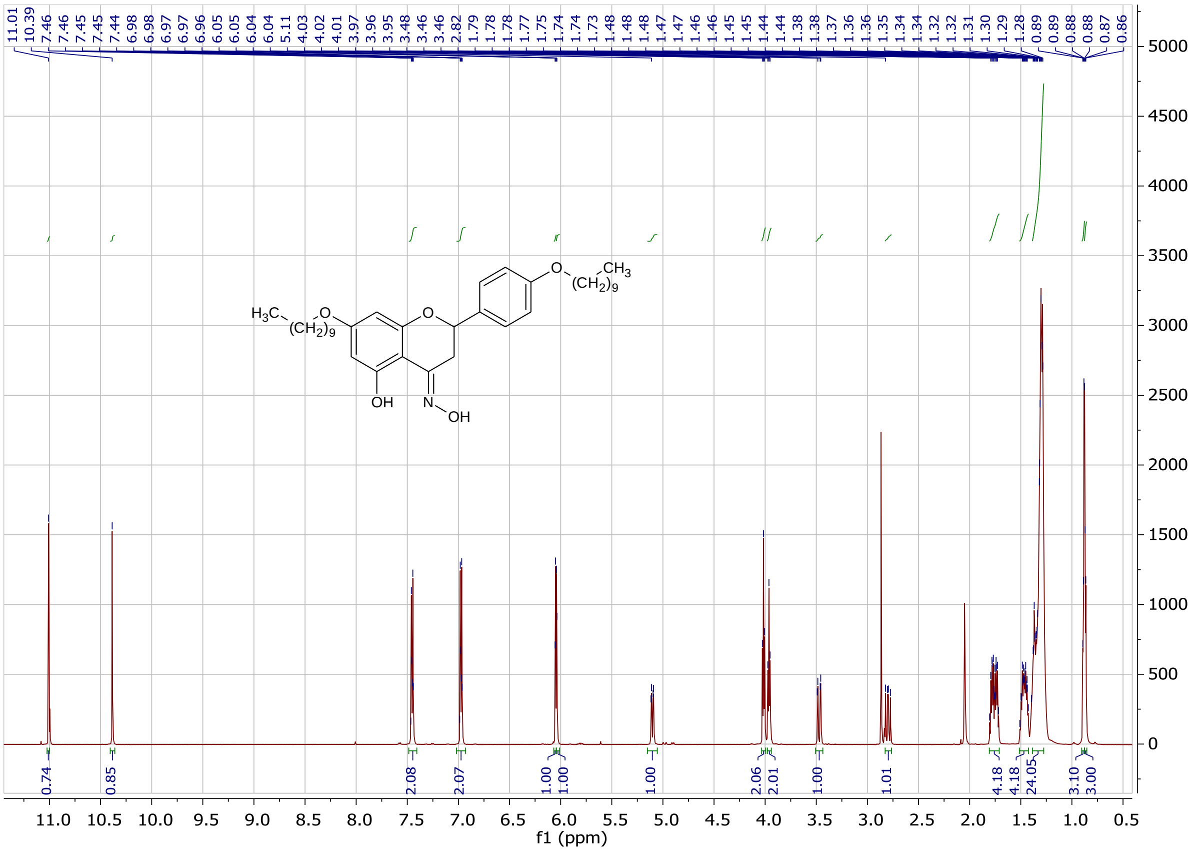

Fig S35. ^{1}H-NMR (600 MHz, $(CD_3)_2CO$) spectrum of 7,4′-Di-*O*-decylnaringenin oxime (**17b**)

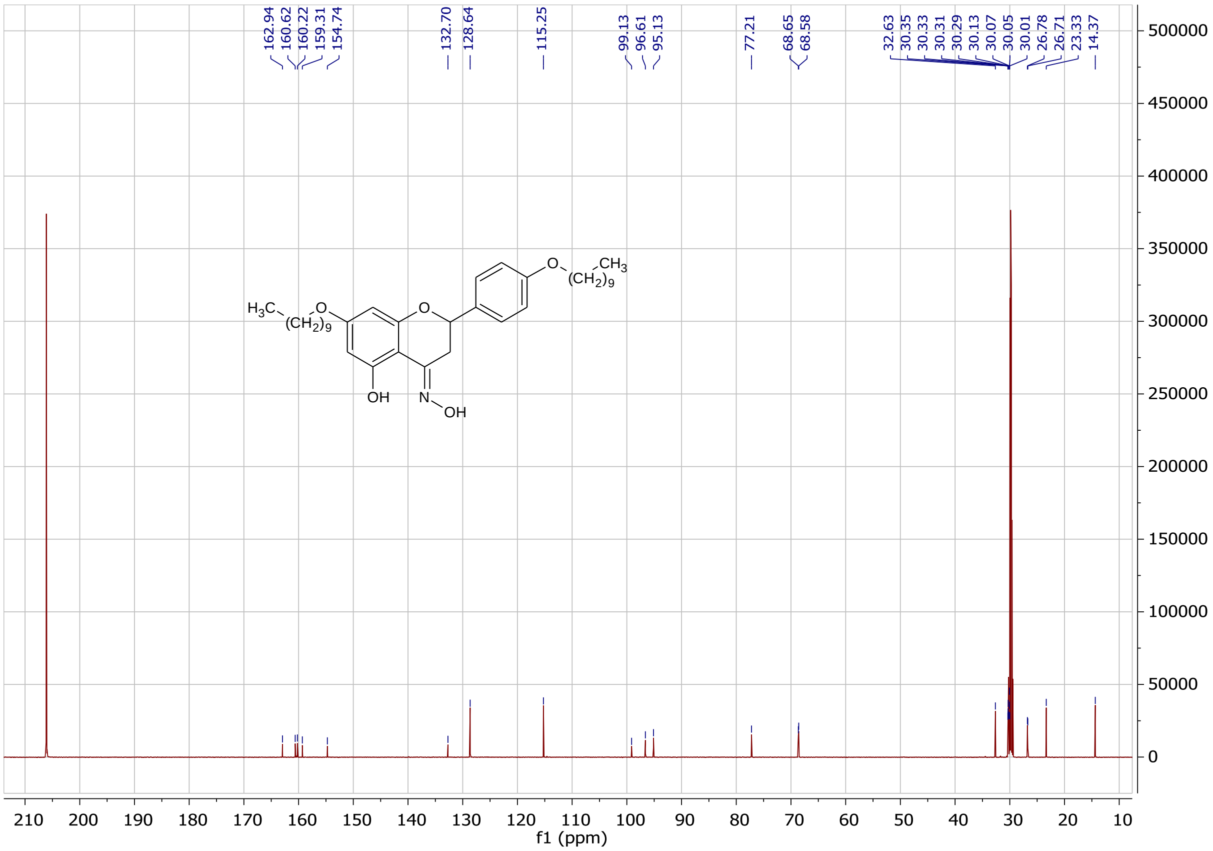

Fig S36. ^{13}C-NMR (150 MHz, $(CD_3)_2CO$) spectrum of 7,4′-Di-*O*-decylnaringenin oxime (**17b**)
